# Molecular Imaging of In Vivo Zinc Distribution Using Nuclear and Magnetic Resonance Imaging

**DOI:** 10.1021/cbmi.5c00264

**Published:** 2026-05-13

**Authors:** Changhua Mu, Xiaoxi Liu, Umama Ali, Peder E. Z. Larson, Sunita P. Ho, Robert R. Flavell

**Affiliations:** † Department of Radiology and Biomedical Imaging, University of California, San Francisco, San Francisco, California 94107, United States; ‡ Helen Diller Family Comprehensive Cancer Center, University of California, San Francisco, California 94158, United States; § Division of Preclinical Simulation and Bioengineering, University of California, San Francisco, San Francisco, California 94143, United States; ∥ Department of Preventive and Restorative Dental Sciences School of Dentistry, University of California, San Francisco, San Francisco, California 94143, United States; ⊥ Department of Urology School of Medicine, University of California, San Francisco, San Francisco, California 94143, United States; # Department of Pharmaceutical Chemistry, University of California, San Francisco, San Francisco, California 94158, United States

**Keywords:** altered zinc homeostasis, zinc-associated diseases, radioactive zinc, nuclear zinc biosensors, zinc-responsive MRI imaging contrasts, nuclear imaging, magnetic resonance imaging (MRI), relaxivity, chemical exchange saturation transfer, nuclear hyperpolarization

## Abstract

Zinc, a ubiquitous trace element, is essential to numerous biological processes, including enzymatic catalysis, gene expression, and cellular signaling. Precise zinc homeostasis, maintained by a complex interplay of transporters, storage proteins, and regulatory mechanisms, is critical for cellular function and organismal health. Disrupted zinc homeostasis has been implicated in the pathophysiology of diabetes, neurodegenerative disorders, and cancers, through alterations in zinc-dependent signaling pathways, oxidative stress responses, and apoptosis mechanisms. Thus, zinc biodistribution is a potential biomarker enabling improved detection of zinc-related pathologies, assessment of disease prognosis, and development of more targeted prevention strategies. While fluorescence techniques have advanced our understanding of cellular zinc, their application in human studies remains restricted. This highlights the need for zinc-specific biosensors that can be integrated with clinical imaging technologies, such as Positron Emission Tomography (PET), Single Photon Emission Computed Tomography (SPECT), and Magnetic Resonance Imaging (MRI), to enable in vivo visualization of zinc distribution and dynamics. This review describes the mechanistic understanding of zinc-associated diseases, details the design principles of molecular zinc biosensors (including radiopharmaceuticals and MRI contrast agents), and identifies the challenges and future directions for developing more specific and sensitive probes. For nuclear imaging (PET and SPECT), this includes probes utilizing radioactive zinc ions ^65^Zn, ^62^Zn, and ^63^Zn, and zinc chelators labeled with isotopes like ^18^F, ^11^C, and ^125^I. For MRI, detection mechanisms leverage relaxivity (*r*_1_), Chemical Exchange Saturation Transfer (CEST), and hyperpolarization technologies. These imaging modalities exhibit the potential of bridging the gap between cellular and systemic zinc organ-level dynamics, offering a “horizontal” perspective on disease pathogenesis and facilitating precision medicine.

## Graphical abstract

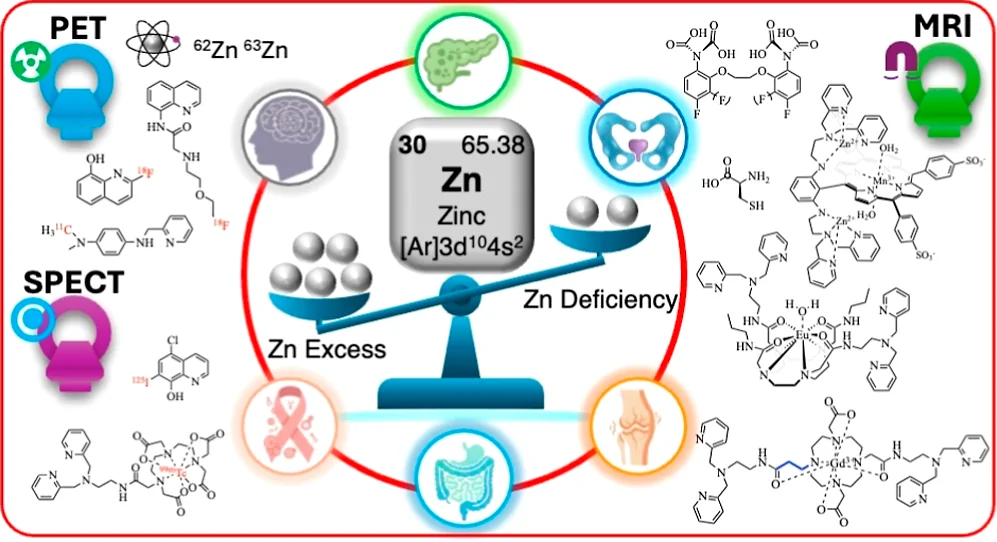

## 1 Introduction

Zinc ion (Zn^2+^) is a ubiquitous micronutrient essential for maintaining health and normal physiological functions.^1,2^ It acts as a vital cofactor for over 300 enzymes involved in diverse biochemical pathways, including DNA, RNA, and protein synthesis, cell division, immune system support, antioxidant defense, nutrient metabolism, neurotransmission, and hormone regulation.^3,4^ An adult human body contains about 2–4 g of zinc, with the majority of distribution in bone and skeletal muscle (86%), skin (4.2%), liver (3.4%), and smaller amounts in other organs.^5^ The discrepancies in zinc levels among various organs implicate the existence of intricate homeostatic mechanisms that precisely regulate the requisite amount of zinc in each organ to support its distinct physiological functions, such as the secretory tissues of the pancreas, prostate, and mammary glands. Cellular zinc homeostasis involves the interplay of complex biochemical processes that control zinc influx and efflux via zinc transporters, and zinc sequestration through binding proteins within cellular organelles to maintain precise zinc concentrations for cellular functions.^6^ There are two families of zinc transporters: the solute carrier 30A (SLC30A) family (ZnT 1–10), which exports zinc out of the cytoplasm, and the SLC39A family (Zrt- and Irt-like proteins, ZIP 1–14), which import zinc into the cytoplasm.^7,8^ Additionally, zinc-binding proteins, such as metallothioneins (MTs), bind, store, and release zinc as needed (Figure 1).^9^ Consequently, dysregulation of cellular zinc homeostasis can reflect the development of various physiological disorders.

**Figure 1:**
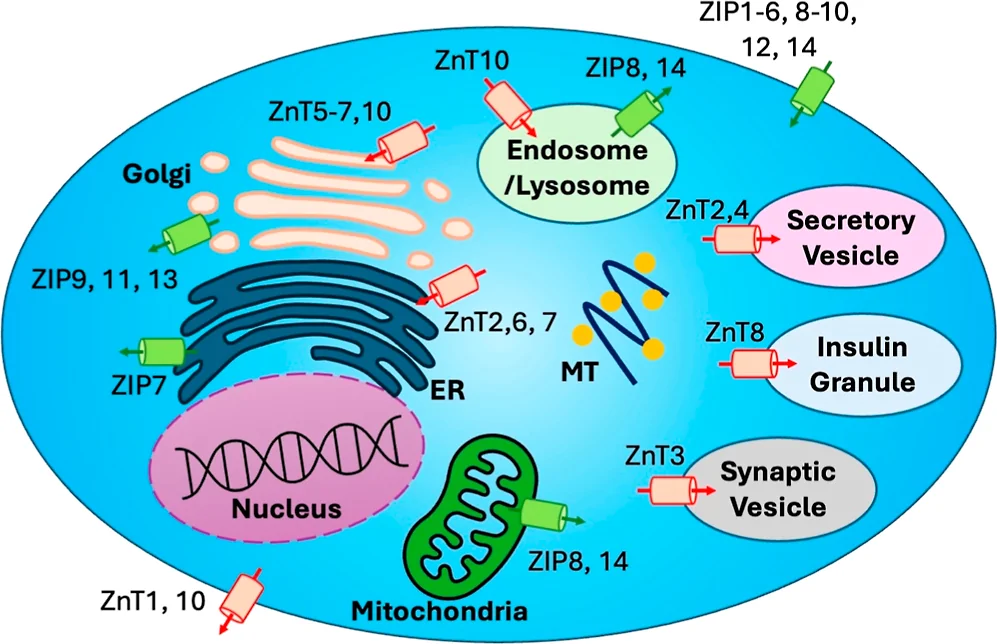
Intracellular regulation of zinc homeostasis. Zinc uptake and efflux are coordinated by the zinc transporter families ZIP (SLC39A) and ZnT (SLC30A), together with metallothioneins (MTs). ZIP transporters mediate zinc influx from the extracellular space or intracellular stores into the cytosol. In contrast, ZnT transporters reduce cytosolic zinc by exporting it out of the cell or sequestering it into organelles. MTs act as dynamic zinc buffers, binding excess zinc and releasing it when needed. These coordinated mechanisms maintain zinc homeostasis, which is essential for enzymatic activity, signaling, and overall cellular function.

The pathologic induction of aggressive diseases, such as prostate cancer, breast cancer, diabetes, pancreatitis, and neurodegenerative diseases, is often accompanied by alterations in zinc levels. For example, while healthy prostate cell cytoplasm and prostatic fluid contain higher zinc concentrations, these levels can decrease significantly, by about 10-fold, in dedifferentiated prostate cancer.^10–12^ Disrupted cellular zinc metabolism and signaling could be etiological factors in these diseases.^13^ Furthermore, dysfunctional changes in zinc levels involved in the biochemical processes could potentially lead to an imbalanced cellular redox status, which could either elevate oxidative stress, causing damage to lipids, proteins, and nucleic acids, or compromise the defense mechanisms (antioxidant actions) against oxidative stress.^14–16^

Altered zinc homeostasis is a biomarker for various diseases, offering significant clinical value for improving early detection, prognosis, and implementation of preventative actions.^17–19^ However, our current understanding of these roles is largely derived from static measurements, such as ex vivo tissue analysis via atomic spectrometry-based techniques like inductively coupled plasma mass spectrometry (ICP–MS), or optical imaging using fluorescent sensors for elemental mapping.^20^ While these traditional techniques provide high-sensitivity snapshots of total or labile zinc concentrations, they are limited in their ability to capture the rapid kinetic flux and spatiotemporal dynamics of zinc as a signaling ion in living organisms. Furthermore, despite the advanced development and ongoing clinical translation of molecular imaging agents, the clinical analysis still relies on measurements from easily accessible tissues, such as blood and biopsies. These methods often serve as poor proxies for both systemic and organ-specific dynamics, as they fail to reflect the complex redistribution of zinc that occurs during disease progression. Consequently, a compelling clinical argument for noninvasive zinc imaging with modalities like positron emission tomography (PET), single-photon emission computed tomography (SPECT), and magnetic resonance spectroscopy/imaging (MRS/I), has yet to be established. To address this gap, this review focuses on studies of biocompatible zinc molecular imaging sensors evaluated in vivo or in humans. Our aim is to evaluate the novelty of their design, analyze their in vivo zinc imaging mechanisms in systemic organs, and provide insights into future directions in both molecular design and clinical translation.

The in vivo imaging of intracellular zinc faces two significant challenges. First, designing suitable zinc biosensors for in vivo imaging is challenging. These biosensors should possess high zinc specificity and targetability, low cytotoxicity, excellent cell membrane permeability, and rapid zinc-binding kinetics. Additionally, upon binding with zinc, the biosensors should exhibit a specific mechanism of forming contrast, such as “switch-on” contrast for zinc-responsive MRI or extended intracellular retention time for nuclear imaging approaches. An efficient strategy to address these challenges is leveraging insights from well-established optical zinc probes. Researchers have developed a wide array of novel optical intracellular zinc probes and methods for quantitative bioimaging of zinc homeostasis in cell lines under microscopy.^21,22^ However, issues such as fluorescence probe photobleaching, high background noise due to light scattering, limited tissue penetration depth, and restricted lateral range present significant limitations for in vivo studies.^23,24^ Nonetheless, these studies have attained the desired traits in designing zinc-chelating structures, providing zinc-specific chelating scaffolds for chemical modification to PET, SPECT, and MRI contrast agents to achieve in vivo zinc sensing and imaging. The additional limitation is the low intracellular concentration of free zinc in most tissues, which do not actively participate in zinc metabolism and functionality, as it is inherently as low as picomolar. Compared to the fluorescence study conducted under optical microscopy, the clinical imaging techniques lack the same high sensitivity and resolution. Thus, in vivo zinc imaging studies predominantly focus on zinc-rich and secretory organs, such as the pancreas, prostate gland, mammary gland, and hippocampus in the brain, where the degree of alteration in zinc homeostasis significantly differs throughout the initiation, exacerbation, and later stages of the diseases.

In this review, we begin by elucidating the roles of zinc in maintaining health and the correlation of altered homeostasis with diseases’ initiation and progression. Subsequently, we thoroughly analyze the ingenuity in the design of zinc contrast agents and their utilization in nuclear imaging (PET and SPECT) and zinc-responsive MRS/I, with assessments of the underlying mechanisms. In nuclear imaging, we discuss two types of contrast agents: radioactive zinc ions, such as ^65^Zn, ^62^Zn, and ^63^Zn, which are used to investigate whole-body zinc ion distribution and trafficking, and radiolabeled zinc-specific chelating agents for PET and SPECT imaging, which exhibit the potential to image endogenous zinc pools at an organ level and quantitatively measure their concentrations. In MRS/I imaging, we evaluate the chemical structural design of zinc-responsive contrast agents and their imaging mechanisms for three imaging methods: relaxivity (*r*_1_), chemical exchange saturation transfer (CEST), and nuclear hyperpolarization (HP). This review focuses on discussing the in vivo results of the sensors, particularly their capability to detect altered zinc homeostasis in organs and its correlation with disease models. It also emphasizes the importance of sensor synthesis and in vitro evaluation as a foundation for establishing structure-activity relationships to optimize zinc sensing efficiency, ultimately benefiting precision medicine.

## 2 Zinc and Diseases

### 2.1 Zinc and Pancreatic Diseases

Pancreatic diseases afflict over 10% of the world population.^25^ In terms of physiological function and cellular origin, they can be categorized into those affecting the exocrine and endocrine pancreas. The exocrine compartment, comprising the majority of the organ’s mass, is primarily involved in the synthesis and secretion of digestive enzymes into the gastrointestinal tract; consequently, diseases in this category include acute and chronic pancreatitis, exocrine pancreatic insufficiency (EPI), and pancreatic adenocarcinoma. In contrast, the endocrine pancreas consists of the Islets of Langerhans, which regulate systemic glucose homeostasis through the secretion of hormones like insulin and glucagon. Pathologies of the endocrine system typically manifest as metabolic disorders, most notably Type 1 and Type 2 diabetes mellitus, as well as rarer pancreatic neuroendocrine tumors (pNETs).^26^

Despite their different clinical presentations, these disorders share a common underlying feature—the dysregulation of zinc homeostasis—which disrupts zinc’s essential functions in the pancreas, including its roles in insulin synthesis, secretion, and antioxidant defense. This imbalance leads to β-cell dysfunction in diabetes, zinc deficiency-induced exocrine insufficiency in chronic pancreatitis, and altered tumor biology in pancreatic cancer, underscoring zinc’s central and interconnected mechanistic role in pancreatic health and disease.

#### 2.1.1 Zinc and Diabetes Mellitus

Diabetes mellitus is characterized by a chronic metabolic increase in blood glucose concentration (hyperglycemia) due to insufficient insulin secretion or impaired action of insulin, the hormone responsible for regulating the concentration and utilization of glucose in the blood plasma.^27,28^ There are two types of diabetes with distinct etiologies. Type 1 diabetes is an autoimmune disease triggered by an autoimmune response against pancreatic β-cells, leading to impaired insulin secretion and subsequent hyperglycemia.^29^ In contrast, hyperglycemia in type 2 diabetes results from a combination of relative insulin deficiency and insulin resistance in various tissues, including adipose and muscle tissues. The failure of β-cell function in the pancreas and a reduction in their mass contribute to inadequate insulin production.^30,31^ Furthermore, excessive insulin production by pancreatic islets in response to hyperglycemia leads to hyperinsulinemia, which triggers a downregulation of cellsurface insulin receptors and exacerbates the condition.^32^

In the 1930s, Scott et al. discovered the presence of zinc in the insulin complex.^33^ Subsequently, they identified a link between the development of diabetes and a significant decrease in zinc content (about 50%) in the pancreas of diabetic patients.^34^ Pancreatic β-cells perform essential functions in the biosynthesis, storage, and secretion of insulin, with zinc as both a necessary material and a structural component in these processes.^35^ These cells harbor the highest zinc concentrations, with approximately 70% localized within insulin secretory granules, maintaining a concentration range of 10–30 mM/kg.^36,37^ This high concentration is essential for the zinc ions to coordinate with insulin monomers, forming the stable, crystalline insulin hexamers required for storage.^38^ Extracellular labile zinc enters the cytosol via ZIP4 transporters on the cell membrane,^39^ followed by uptake into intracellular secretory granules through ZnT8 on their membranes.^40,41^ During insulin synthesis (Figure 2), proinsulin is transported to the Golgi apparatus and then incorporated into secretory granules, where two zinc ions coordinate with two insulin dimers to form crystalline insulin, which then combines with an additional insulin dimer to produce zinc-containing hexameric insulin complexes.^42^ Upon glucose stimulation, healthy β-cells exocytose dense-core granules that store insulin as zinc-coordinated crystalline hexamers; once exposed to the extracellular milieu these hexamers dissociate rapidly on the order of seconds to monomeric insulin and free zinc ions,^43^ leading to a transient local rise in extracellular zinc concentration from very low baseline free levels (pico- to low-nanomolar) to high-nanomolar or low-micromolar concentrations in the pericellular space, with some measurements reporting transient peaks reaching tens to a few hundred micromolar immediately at the cell surface.^44^

**Figure 2:**
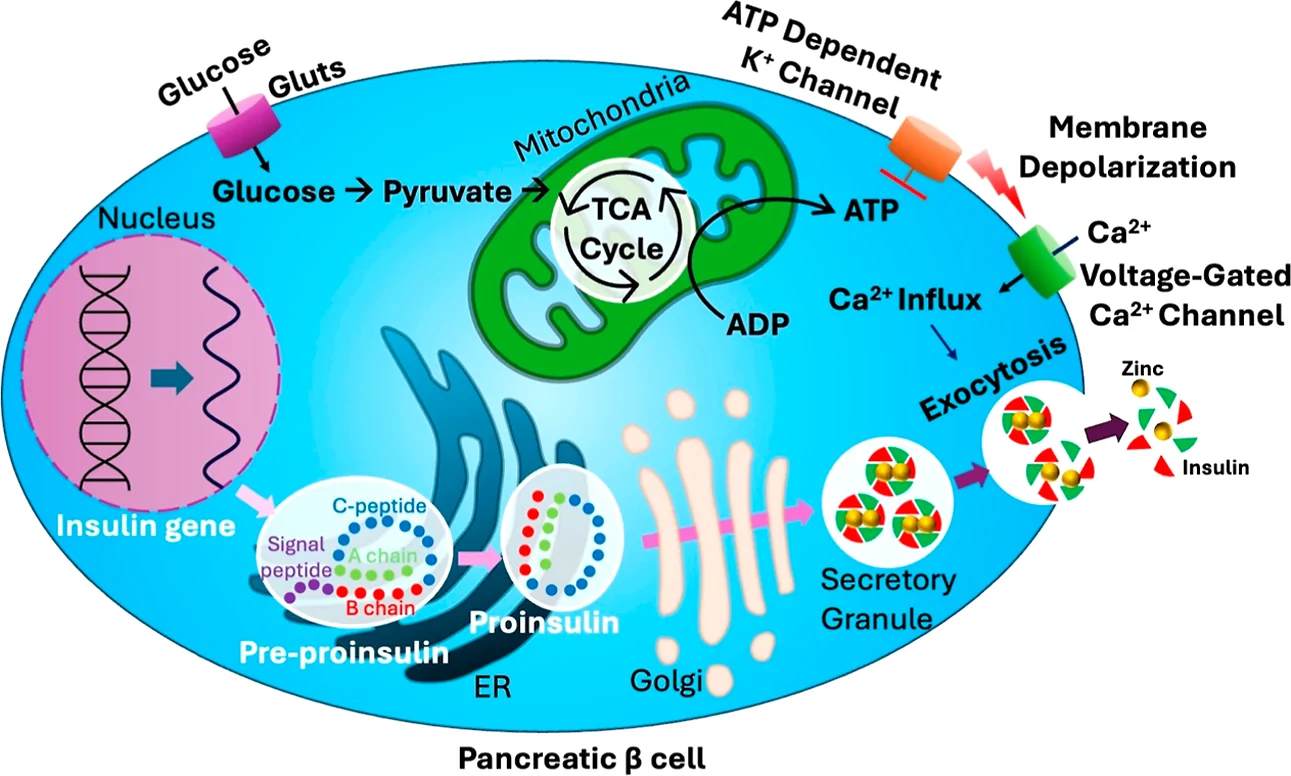
Insulin biosynthesis and secretion in pancreatic β-cells. The process of insulin formation and secretion begins in the nucleus of pancreatic beta cells, where the insulin gene is transcribed. The resulting mRNA is translated on the rough endoplasmic reticulum (ER) into preproinsulin, which is then cleaved to form proinsulin. This prohormone is transported to the Golgi apparatus and packaged into secretory granules, where it is cleaved into mature insulin and stored as crystalline hexamers. Upon glucose stimulation, glucose is metabolized into pyruvate, which enters the mitochondria and fuels the TCA cycle, increasing the intracellular ATP/ADP ratio. This change triggers the closure of ATP-sensitive potassium channels, leading to membrane depolarization. The depolarization opens voltage-gated Ca^2+^ channels, and the resulting influx of calcium ions serves as the final trigger for the granules to undergo exocytosis, releasing insulin hexamers into the bloodstream, where they rapidly dissociate into active monomers and free zinc ions.

Pancreatic β-cells in both type 1 and type 2 diabetes contain significantly less zinc content than healthy cells. During hyperglycemia, excessive glucose metabolism drives an overproduction of insulin and accelerates mitochondrial oxidative phosphorylation in β-cells, leading to increased generation of reactive oxygen species (ROS), particularly superoxide anions. This metabolic overdrive is driven by enhanced glucose oxidation through the tricarboxylic acid cycle and subsequent electron leakage from the mitochondrial electron transport chain. In addition, hyperglycemia activates alternative pathways—such as the polyol and hexosamine pathways, protein kinase C signaling, and advanced glycation end-product (AGE) formation—that further amplify oxidative and inflammatory stress. The cumulative oxidative burden damages β-cell membranes, DNA, and insulin secretory machinery, impairing function and contributing to progressive β-cell exhaustion in diabetes.^45^ Consequently, impaired pancreatic β-cells exhibit an exhausted insulin secretory capacity, which results in a reduced and delayed extracellular zinc response upon glucose stimulation, reflecting decreased secretory activity over time.^46^

Zinc maintains the delicate balance of reduction–oxidation (redox) reactions essential for sustaining normal biological functions. It exerts its antioxidant and anti-inflammatory effects in various pathways, including activating antioxidant proteins and enzymes, such as glutathione (GSH) and catalase, and stabilizing protein sulfhydryls against oxidation. Importantly, zinc acts as a structural stabilizer and a competitive antagonist to attenuate cellular site-specific oxidative injury by inhibiting transition metal-catalyzed reactions, particularly those involving copper and iron, at specific binding sites. The underlying mechanism relies on GSH’s high affinity for zinc over iron and copper. This preferential binding, which relates to the specific atomic structure and sulfhydryl groups of GSH, makes zinc a key factor in protecting GSH-dependent redox cycling and preventing site-specific Fenton chemistry.^14^

Additionally, zinc reduces NF-κB activation, leading to increased expression of A20 and PPAR-α, two zinc finger proteins associated with anti-inflammatory properties.^47^ Furthermore, zinc-binding and oxidative stress related sequestering proteins, metallothioneins (MTs), serve as potent electrophilic scavengers and provide cyto-protection, exhibiting approximately 50 times greater activity than GSH.^48^ Consequently, when MTs are deficient due to low zinc availability, high oxidative stress, or pathological impairment, elevated oxidative stress can activate NF-κB and its downstream pathways, resulting in enhanced humoral responses.^49^

#### 2.1.2 Zinc and Pancreatitis

Moreover, zinc deficiency is potentially linked to chronic pancreatitis—a chronic inflammation with progressive fibrosis of the pancreas, ultimately resulting in pancreatic exocrine insufficiency, which is characterized by deficiencies of the enzymes secreted from the pancreas due to its malfunction caused by diseases, including pancreatic cancer.^50^ The resulting condition leads to maldigestion, which subsequently causes malnutrition, specifically deficiencies in fat-soluble vitamins, antioxidants, and other micronutrients.^51,52^ Similar to the cosecretion of zinc and insulin, stimulation with secretin and cholecystokinin results in the secretion of zinc into pancreatic juice with amylase and trypsin at higher concentrations than in patients with chronic pancreatitis. Therefore, measuring zinc levels in pancreatic or duodenal juice could be an alternative to enzyme assays for diagnosing chronic pancreatitis.^53^

#### 2.1.3 Zinc and Pancreatic Cancer

Pancreatic cancer highlights the functional dichotomy of the organ’s compartments: exocrine pancreatic cancer, primarily pancreatic ductal adenocarcinoma (PDAC), arises from the enzyme-secreting tissues and represents the predominant clinical form, while endocrine pancreatic cancer consists of pNETs originating from the hormone-producing Islets of Langerhans.^54^ Both malignancies are characterized by the dysregulation of zinc and its transporters, which is closely linked to impaired glycemic control and carcinogenesis.^55,56^

In exocrine tumors, a marked decrease in intracellular zinc is often observed; conversely, in endocrine tumors, zinc levels are intrinsically tied to the specialized secretory machinery of the islet cells. This systemic imbalance exacerbates oxidative stress, impairs β-cell function, and disrupts insulin secretion, creating a metabolic environment that may promote tumor initiation and progression. Clinically, zinc deficiency in patients, particularly those with pancreatic exocrine insufficiency, correlates with a poor prognosis, including suboptimal surgical outcomes and reduced overall survival rates. Such evidence underscores the necessity of compartmentalized approaches when studying zinc-based biomarkers or therapeutic targets in pancreatic oncology.

### 2.2 Zinc and Prostate Cancer

Prostate cancer is characterized by high morbidity and mortality, with 313,780 new cases and 35,770 deaths estimated in the U.S. in 2025,^57^ representing a biologically heterogeneous disease with a broad clinical spectrum, ranging from indolent cases to highly aggressive forms.^58^ Typically, its onset, like benign prostatic hyperplasia (BPH), usually grows slowly and remains confined to the prostate gland without causing noticeable symptoms. However, in certain instances, as it advances, it can become lethally aggressive and spread to other parts of the body. Thus, the evolving nature of prostate cancer emphasizes the importance of precise and timely detection. Zinc can serve as a biomarker for advanced imaging techniques, potentially enabling the detection of aggressive prostate cancer, patient stratification, monitoring of disease progression, and evaluation of therapeutic responses.

Similar to the pancreas, the prostate is an organ known for zinc accumulation. Mawson and Fischer first reported in the 1950s that the prostate contains more zinc than other organs.^59^ In a normal prostate, zinc accumulates intracellularly to a remarkably high concentration of 10–100 mM, the highest amount of zinc in mammalian tissue.^60^ In contrast to pancreatic β-cells that specifically sequester zinc into their secretory granules, prostate cells allocate almost 35% of the total intracellular zinc content within their cytoplasm, where zinc loosely binds with small molecules, like citrate, to sustain essential biological functions.^61^ The prostate accumulates a substantial amount of zinc to optimize male reproductive processes, including spermatogenesis, sperm quality, and fertilization.^62^ Additionally, zinc supports sperm release and motility in the prostatic fluid^63^ and exhibits antimicrobial effects in the prostatic fluid and the prostate.^64,65^

The underlying mechanism responsible for the high intracellular zinc is complex. Recent studies suggest that the upregulation of ZIP1 in prostate cells facilitates zinc accumulation, possibly inhibiting cell growth and promoting net citrate production.^66,67^ The prostate gland’s primary function, particularly the peripheral zone, which constitutes 70% of the prostate, is to produce and secrete exceptionally high levels of citrate. The secretory epithelial cells accumulate a high level of zinc in the prostate at 1018 ± 124 μg/g dry tissue,^68^ which is 3 to 10–fold higher than in other soft tissues, to inhibit the activity of mitochondrial aconitase—a key enzyme in converting citrate to isocitrate (oxidized form of citrate) within the Krebs cycle.^69^ This inhibition influences energy production and metabolic processes in the mitochondria, preventing citrate oxidation and ensuring the substantial provision of citrate for secretion into the prostatic fluid.^70^

The zinc accumulation mechanism becomes impaired early in prostate cancer development and persists throughout progression to castration-resistant stages, resulting in a 60–80% reduction in zinc levels (146 ± 10 μg/g dry tissue).^10,11,68,71^ The mechanisms of decreased zinc content in prostate cancer are complex and remain incompletely understood, involving alterations in zinc transport, metabolism, and storage pathways. This reduction stems from downregulated expression of ZIP1, a key zinc transporter.^66,72^ Consequently, crucial cellular processes are disrupted, including energy metabolism (increased citrate oxidation), apoptosis (programmed cell death), and cell cycle regulation. Furthermore, altered zinc levels also affect key signaling pathways, such as androgen receptor signaling, which is pivotal for prostate cell growth and function.^73^ Although the precise mechanisms and their influence on cancer initiation and progression require further investigation, these observations demonstrate the crucial role of zinc in prostate cancer pathogenesis.

### 2.3 Zinc and Neurodegenerative Disorders

Neurodegenerative disorders are diseases marked by the progressive degeneration of neurons in the brain and spinal cord, leading to a gradual loss of function and a spectrum of symptoms, including cognitive decline, movement impairments, and sensory disturbances. Conditions such as Alzheimer’s disease (AD), Parkinson’s disease, Huntington’s disease, and amyotrophic lateral sclerosis (ALS) profoundly impact patients’ quality of life and pose significant challenges to healthcare systems due to their complexity and long-term care demands.^74^ Among these, AD, the most common form of dementia, affects millions worldwide and is rapidly increasing in prevalence with the aging population. This rise imposes a substantial societal burden, including escalating healthcare costs, caregiver strain, and an impact on the overall economy. Families often face immense emotional and financial challenges, while patients and caregivers alike endure social isolation, further diminishing quality of life.^75^

AD is neuropathologically defined by the accumulation of senile plaques and neurofibrillary tangles within brain tissue.^76^ These hallmark features arise from the excessive aggregation of misfolded β-amyloid (Aβ), hyperphosphorylated tau proteins, and other mechanisms, leading to cytotoxicity, disruption of neuronal cytoarchitecture, and subsequent neuronal death, culminating in a progressive decline in brain function. Other key pathological characteristics of AD include neuroinflammation, cholinergic deficits, impaired glucose metabolism, and synaptic dysfunction.^77^ Additionally, elevated levels of metal ions, such as iron, copper, and zinc, have been detected in amyloid plaques, with concentrations of iron and zinc reaching as high as 1 mM in the vicinity of these deposits. These ions are believed to exacerbate Aβ pathogenicity, further contributing to the disease progression.^78,79^ Notably, only zinc and copper have been found to directly coordinate with Aβ in the AD-affected brain, and metal chelators have emerged as therapeutics to target these metal ions, showing the ability to dissolve Aβ deposits and demonstrating the promise of mitigating disease progression.^80,81^

Specifically, zinc plays a multifaceted role in the pathophysiology of AD, influencing Aβ aggregation, synaptic function, and neuroinflammation. Zinc ions can directly bind to Aβ, promoting its aggregation into plaques, a hallmark of AD pathology. Elevated zinc levels, particularly in amyloid plaques, suggest a link between zinc homeostasis and Aβ deposition. Dysregulated zinc signaling has also been implicated in synaptic dysfunction, as zinc is essential for normal neurotransmission and synaptic plasticity, both of which are impaired in AD. Furthermore, zinc imbalance may exacerbate neuroinflammation by modulating microglial activation and inflammatory cytokine production, both of which are known to contribute to neurodegeneration.^82^ Thus, zinc appears to be intricately connected to multiple pathways underlying AD progression, positioning it as a potential target for therapeutic interventions aimed at restoring zinc balance and mitigating disease progression.

### 2.4 Zinc and Other Diseases

In addition to the aforementioned diseases, in which the feasibility of imaging zinc homeostasis has been actively investigated using noninvasive and human clinical imaging modalities, a substantial number of aggressive conditions related to zinc metabolism remain unexplored, such as breast cancer, osteoarthritis, gastrointestinal cancers, and genetic disorders.^13^ For instance, breast cancer remains a significant global health concern, with triple-negative breast cancer representing a particularly aggressive subtype that accounts for 10–20% of breast cancers. Compared to benign breast tissue, breast cancer cells exhibit significantly higher levels of zinc accumulation (∼13.3 μg/g tissue) due to the overexpression of specific zinc transporters in malignant cells.^83^ Qu et al. conducted a comprehensive review regarding the zinc-related metabolism in breast cancer.^84^

Similarly, Osteoarthritis (OA) is a leading cause of disability among older adults, impacting approximately 528 million individuals worldwide according to a report on the Global Burden of Diseases (2019), indicating an increase of 113% since 1990.^85^ The prevalence of OA is still increasing and projected to affect 25% of U.S. adults by 2030,^86^ emphasizing the growing significance of OA as a health concern. OA is a degenerative joint disease characterized by the irreversible degradation of cartilage; however, effective OA management through both nonsurgical and surgical treatments can effectively alleviate pain, enhance joint flexibility, and improve overall quality of life for patients. Thus, the capability of noninvasively assessing articular cartilage degeneration, involving simultaneous imaging of both tissue alteration at the molecular level and compositional and morphological damage, exhibits great clinical value in enabling early detection and prevention. Clinical studies revealed significantly elevated serum zinc levels in patients with OA^87^ and specific accumulation of zinc in the tidemark region of articular cartilage,^88,89^ which may be attributed to the specific upregulation of ZIP8 in chondrocytes, as demonstrated in studies of humans and mice.^88^

Altered zinc levels are implicated in gastrointestinal cancers.^84^ For instance, colorectal cancer exhibits a significant 66% decrease in mean tissue zinc in malignant versus noncancerous tissue from the same patients.^90^ Similarly, gastric cancer shows reduced zinc levels from early (78.7 ± 29.6 μg/dl) to advanced stages (66.9 ± 17.8 μg/dl).^91^ Esophageal cancer also presents with lower zinc in patients’ tissue (81 μg/g) compared to control subjects (97 μg/g [dry weight]).^92^

Furthermore, the clinical impact of zinc dysregulation is clearly correlated with genetic disorders involving the SLC30 (ZnT) and SLC39 (ZIP) transporter families. These hereditary disorders, such as Acrodermatitis Enteropathica (ZIP4), Spondylocheirodysplastic Ehlers–Danlos Syndrome (ZIP13), Transient Neonatal Zinc Deficiency (ZnT2), and Parkinsonism/Manganism (ZnT10), exemplify how specific transporter dysfunctions drive complex and systemic pathologies. The most prominent example, Acrodermatitis Enteropathica, arises from SLC39A4 (ZIP4) mutations that impair intestinal zinc absorption, leading to severe dermatitis and immune dysfunction. Similarly, the “lethal milk” mouse model (ZnT4) demonstrates how defects in zinc secretion can cause fatal, organ-specific deficiencies. Collectively, these cases suggest that zinc transporter dysregulation plays a foundational role in a broader spectrum of human diseases than previously recognized.^93^

The above discussion highlights the necessity and novelty of developing zinc-specific biosensors for using noninvasive clinical imaging technologies, particularly the PET, SPECT, and MRI contrast agents, to investigate the dynamic role of zinc across a broad spectrum of diseases. These approaches make it possible to detect and characterize pathological processes at the molecular level, often preceding anatomical changes or the onset of clinical symptoms. For instance, imaging can capture the decline of zinc in prostate cancer as well as its dysregulation in breast and gastrointestinal cancers, establishing zinc as a specific biomarker for diagnosis and disease characterization. In neurodegenerative disorders such as Alzheimer’s disease, zinc imaging provides critical insights into dyshomeostasis associated with neuronal injury and diagnosable cognitive decline. Beyond oncology and neurology, it offers functional assessment in diabetes through real-time tracking of zinc release from pancreatic β-cells, and in osteoarthritis by visualizing zinc fluctuations linked to inflammation and cartilage degradation. By enabling longitudinal, patient-specific evaluation of zinc imbalance and therapeutic response, in vivo zinc imaging exhibits transformative potential for advancing early detection, guiding interventions, and driving the era of precision medicine.

## 3 Nuclear Imaging Technologies for In Vivo Zinc Distribution and Imaging

### 3.1 Radioactive Zinc for In Vivo Biodistribution and Whole-Body Trafficking Studies

Zinc (Zn) has five stable isotopes, with ^64^Zn being the most abundant (48.6%), followed by ^66^Zn (27.9%), ^68^Zn (18.8%), ^67^Zn (4.1%), and ^70^Zn (0.6%). Additionally, several radioactive isotopes, most notably ^65^Zn (γ-emitter), ^62^Zn (positron emitter), and ^63^Zn (positron emitter), have been used for tracking in vivo zinc distribution. As early as the 1940s, Sheline et al. conducted pioneering research on the distribution, metabolism, and excretion of ^65^Zn in dogs and mice.^94,95^ Over the following three decades, researchers extensively used ^65^Zn, either as ^65^ZnCl_2_ or amino-acid-bound complexes, in various in vivo studies. The outcomes observed in human studies are particularly noteworthy, revealing the dynamics of zinc distribution, trafficking, metabolism, and turnover within human organs, including diseased ones.^96–103^

Despite emitting gamma photons and low-abundance positrons, ^65^Zn proves unsuitable for whole-body in vivo imaging due to safety concerns associated with its long half-life (244 days), posing challenges such as a high absorbed radiation dose and subsequent difficulties in handling radioactive waste. Thus, researchers have shifted their preference toward ^62^Zn and ^63^Zn for PET imaging, which has advanced the capability to map dynamic (real-time) information on the trafficking of radionuclides in biological processes throughout the body.

^62^Zn undergoes decay through electron capture and β^+^ emission with a half-life of 9.3 h. In a pioneering study reported in 1977, Yano and Budinger synthesized ^62^ZnCl_2_ and five ^62^Zn-chelated amino acid complexes and administered these formulations into rats, dogs, and monkeys.^104^ Using scintillation and Anger positron cameras, alongside necropsy biodistribution studies, the researchers compared the uptake and clearance of these formulations among various organs, focusing on the liver, pancreas, and prostate across different animal subjects. ^62^Zn demonstrated similar localization patterns with pancreas-to-liver ratios near 1.0 (up to 1.44) and sufficient pancreas and prostate uptake specificity, supporting its feasibility for emission computed tomography. However, ^62^Zn may not be an ideal radionuclide for PET imaging of in vivo zinc trafficking due to its complex decay into the daughter isotope ^62^Cu. At secular equilibrium, the PET signal is dominated by ^62^Cu, which has a high positron branching fraction (98%) compared to the negligible contribution from ^62^Zn (8.2%). Consequently, the 511 keV annihilation photons detected by PET originate primarily from the daughter ^62^Cu rather than the parent ^62^Zn, posing challenges in distinguishing zinc-specific biodistribution from copper-related metabolic pathways.^105^

In 2022, Firth et al. re-evaluated this issue by preparing [^62^Zn]­Zn-citrate for PET imaging of zinc trafficking in vivo.^106^ They leveraged the significant half-life disparity between ^62^Zn (9.3 h) and ^62^Cu (9.7 min) to isolate the ^62^Zn signal. They hypothesized that by 1 h postinjection, the majority of the initial ^62^Cu present in the equilibrium mixture of ^62^Zn and ^62^Cu would have undergone six half-lives, decaying to negligible levels. Based on this calculation, the newly produced in vivo ^62^Cu from ^62^Zn decay would exist at a negligible level (<2% of the total activity) and remain colocalized with the parent ^62^Zn at the tissue of interest. Assuming the in situ produced ^62^Cu does not redistribute prior to decay, the resulting PET data would effectively represent the spatial distribution of ^62^Zn within the target organs. To validate this hypothesis, they purified ^62^Zn from the ^62^Zn/^62^Cu generator in the form of [^62^Zn]­Zn-citrate complex and administered it to female BALB/c mice, followed by PET imaging at 0, 1, 4, and 24 h postinjection. At 1 h postinjection, the images showed the signals in the heart, liver, kidney, and intestines, with excretion via the hepatobiliary system. The biodistribution remained similar at 4 h. However, at 24 h, when the activity cleared from the abdominal organs, the pancreas exhibited high signal intensity. These results are consistent with findings from parallel studies conducted by DeGrado et al. using the ^63^Zn isotope (discussed below).^107,108^ Furthermore, the researchers administered [^64^Cu]­Cu-citrate to the animals. They observed a significant difference in trafficking pathway and biodistribution of ^64^Cu compared to ^62^Zn, with high activity detected in the liver, intestines, salivary glands, and kidney but minimal uptake in the pancreas at 4 and 24 h postinjection (Figure 3a).^106^ This approach, thus, can reliably reflect ^62^Zn trafficking in vivo without interference from ^62^Cu, highlighting its advantages for investigating zinc excretion and redistribution between tissues at later time points, such as 48 h or longer.

**Figure 3:**
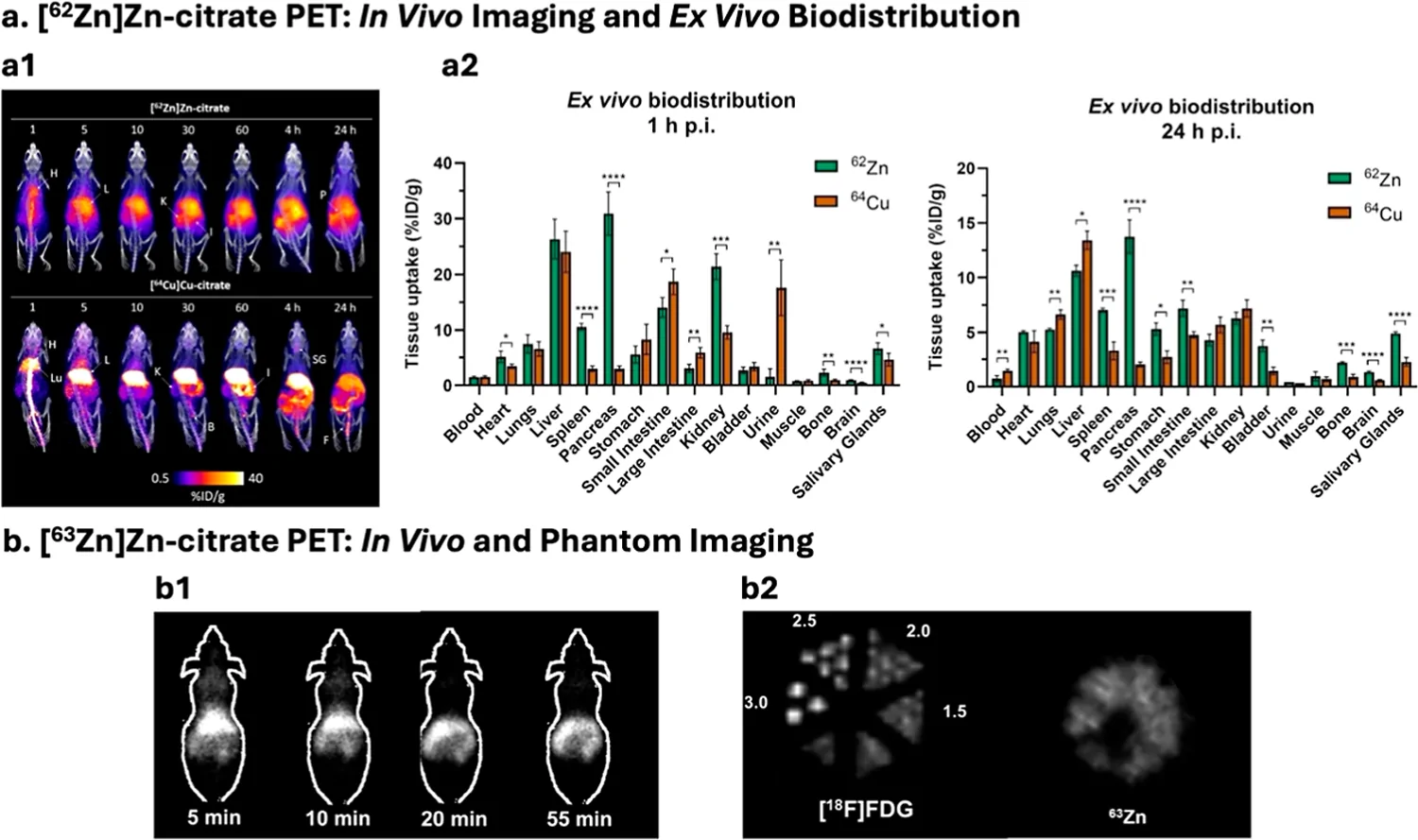
In vivo, ex vivo, and phantom studies characterized the pharmacokinetics and resolution of corresponding radioactive metal ions, ^62^Zn, ^64^Cu, and ^63^Zn. (a) In vivo studies of ^62^Zn and ^64^Cu demonstrate their significantly different biodistribution: (a(1)) PET/CT maximum intensity projections (MIP) show the dynamic distribution of [^62^Zn]­Zn-citrate (top) and [^64^Cu]­Cu-citrate (bottom) in healthy female BALB/c mice (9–11 weeks old) at various time points post intravenous injection (B = bladder, *F* = faeces, H = heart, I = intestines, K = kidney, L = liver, Lu = lungs, *P* = pancreas, and SG = salivary glands), and (a(2)) Ex vivo biodistribution analysis at 1 and 24 h postinjection further validated these findings.^106^ Reproduced with permission from ref 106. Copyright 2022, Oxford University Press; and (b) In vivo ^63^Zn PET imaging shows the dynamic distribution of ^63^Zn, while a phantom study confirmed that ^63^Zn has a relatively lower PET resolution compared to ^18^F: (b1) PET/CT MIP images of [^63^Zn]­Zn-citrate in healthy male B6.SJL mice at various time points post intravenous injection, and (b2) A microresolution phantom was scanned with solutions of either ^63^Zn or ^18^F.^107^ Reproduced with permission from ref 107 Copyright 2014, Society of Nuclear Medicine and Molecular Imaging, Inc.

Compared to ^65^Zn and ^62^Zn, ^63^Zn exhibits a more favorable decay mode (β^+^, 93%, half-life = 38.5 min) for PET imaging of zinc trafficking and biodistribution in vivo. DeGrado et al. (in 2014) successfully developed an approach for producing [^63^Zn]Zn citrate, adhering to stringent quality controls in compliance with current good manufacturing practices (GMPs), and conducted in vivo imaging studies.^107^ They produced ^63^Zn via the ^63^Cu­(*p, n*)^63^Zn reaction by irradiating a ^63^Cu-copper nitrate solution with 14 MeV protons in a low-energy cyclotron, followed by purifying and reformulating ^63^Zn into [^63^Zn]Zn citrate, which was then administered into normal male B6.SJL mice for PET imaging and subsequent ex vivo biodistribution studies (Figure 3b­(1)). The PET images showed increased ^63^Zn uptake in abdominal tissues, particularly in the liver and the gastrointestinal tract. The ex vivo biodistribution provided more detailed information about zinc distribution, quantified using standardized uptake value (SUV)—activity counts per gram of tissue divided by injected total activity counts per mouse weight)—showing ^63^Zn high uptake at 60 min postinjection in the pancreas (SUV, 8.8 ± 3.2), liver (SUV, 6.0 ± 1.9), upper intestine (SUV, 4.7 ± 2.1), and kidney (SUV, 4.2 ± 1.3), and moderate retention in the brain (whole-brain SUV, 0.24 ± 0.09).

In 2016, the same team conducted the first clinical PET imaging studies with ^63^Zn-zinc citrate, involving six healthy elderly participants and six patients diagnosed with Alzheimer’s disease (AD).^108^ The study started with a 30 min dynamic PET imaging of the brain, followed by body imaging and urine and venous blood activity analysis. The dynamic brain PET images demonstrated moderate brain uptake of ^63^Zn, with regional dependencies in pharmacokinetics, showing slower radioactivity clearance in several brain regions of AD patients compared to healthy participants (Figure 4a­(1–3)). Importantly, these regions are consistent with those identified by ^11^C-pittsburgh Compound B for amyloid-β plaque deposition and lower ^18^FFDG uptake. Furthermore, the body PET images revealed the highest ^63^Zn uptake in the liver, pancreas, and kidney, with moderate uptake observed in the intestines, prostate (in males), thyroid, spleen, and pituitary and salivary glands (Figure 4a­(4–6)). The prominent uptake within the digestive system aligns with zinc’s crucial role in gastrointestinal function.^109^ Additionally, two AD patients, also diagnosed with diabetes, exhibited the highest urinary excretion, consistent with their hyperzincuria status.

**Figure 4:**
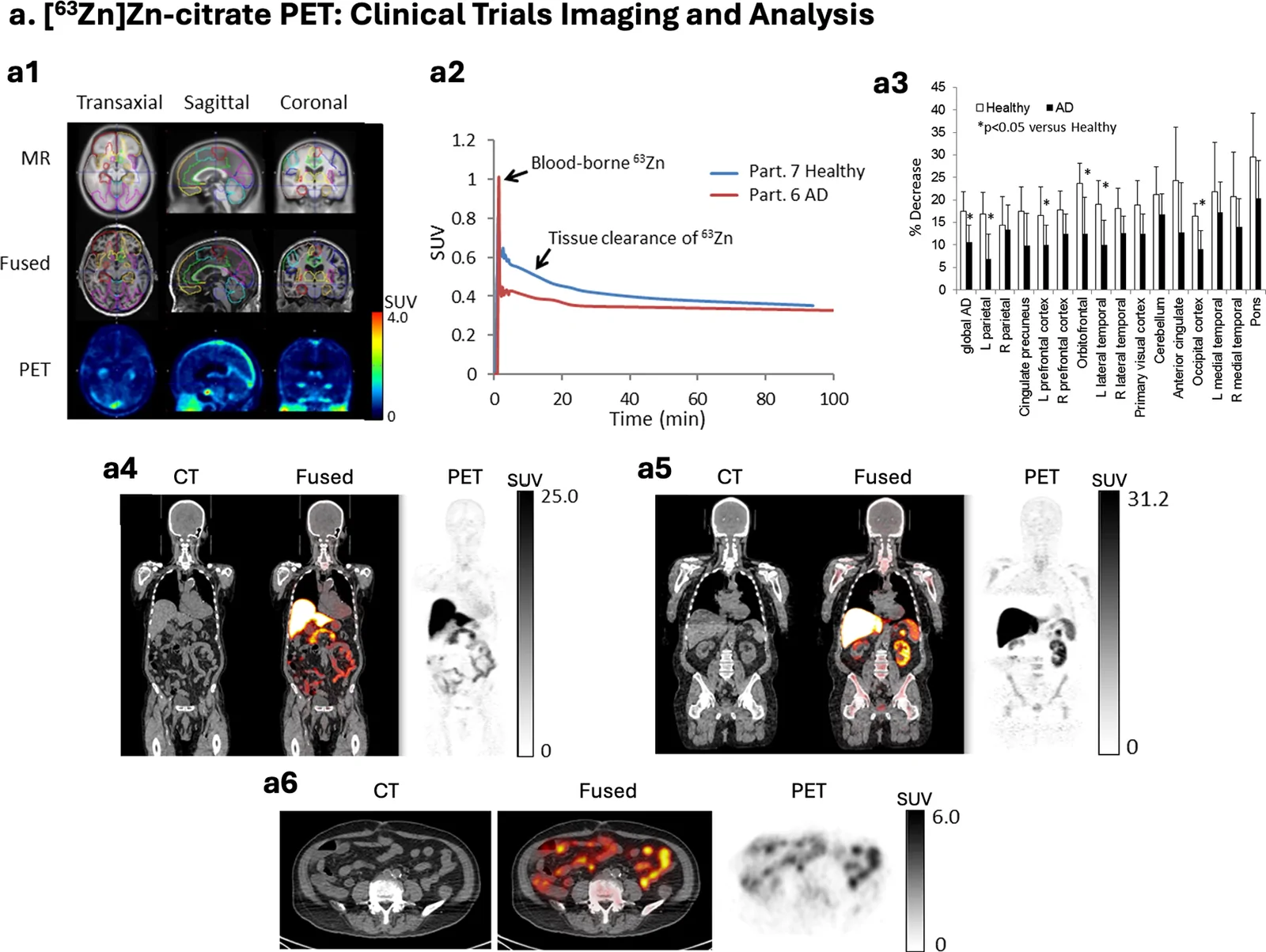
Clinical ^63^Zn PET studies successfully imaged zinc biodistribution in humans. ^63^Zn exhibited high uptake in the liver and moderate uptake in the pancreas, kidneys, spleen, and intestines, with low uptake and slower clearance in the brains of Alzheimer’s disease (AD) patients. (a(1)) Fused PET and *T*_1_-weighted MRI images show the distribution of ^63^Zn in the human brain at 45–50 min postinjection, (a(2)) Time-activity curves for the left prefrontal cortex compare uptake in AD patients and healthy participants, (a(3)) A comparison of regional cerebral clearance of ^63^Zn at 2.5–27.5 min postinjection shows slower regional clearance in the brains of AD patients, which correlated with amyloid-beta pathology, (a(4)) and (a(5)) Whole-body PET/CT images of an AD patient and a healthy elderly participant (45–70 min postinjection) both show prominent hepatic uptake with lower uptake in pancreas, spleen, kidneys and intestines, bone, marrow, and brain. No qualitative differences were observed between them. (a(6)) Abdominal PET/CT images show the intraluminal distribution of ^63^Zn in the duodenum, jejunum, and ileum.^108^ Reproduced with permission from ref 108 Copyright 2014, Copyright 2016, Sage Publications.

Radioactive zinc ions represent a novel approach for the in vivo visualization of zinc’s dynamic transport and trafficking throughout the body, offering the potential to differentiate healthy and diseased tissues. For instance, initial human clinical trials with ^63^Zn PET have demonstrated the clinical relevance, revealing regional variations in zinc clearance kinetics in AD patients that correlated with amyloid-β burden. Furthermore, alternative administration routes, such as oral gavage, could allow radioactive zinc to more accurately reflect the physiological pathway of dietary uptake, potentially opening as-yet unexplored avenues for assessing zinc absorption and transport in gastrointestinal disorders. Clinical translation of oral radiozinc may also be facilitated by less stringent GMP requirements compared to the rigorous restrictions associated with intravenous tracers, though potential applications could be limited by reduced signal intensity due to low intestinal absorption.

The successful clinical trials of ^63^Zn-citrate PET imaging represent an epochal milestone in our ability to probe trace metal homeostasis in humans. Primarily, it has demonstrated—for the first time—a basic biological perspective on the dynamic trafficking of elemental zinc in a clinical setting. However, whether this approach serves as a translational biomarker would depend largely on tissue-specific kinetics. For tissues with slow kinetics, such as muscle and bone, the radiozinc signal decays before reaching equilibrium, meaning the PET data reflect immediate transport flux rather than lifetime homeostatic accumulation. Conversely, in fast-kinetic tissues like the pancreas or liver, the correlation is much stronger, allowing the tracer to serve as a more direct biomarker of total zinc concentration. Thus, while it is an unparalleled tool for mapping dynamic flux, its translational utility as an in vivo zinc-trafficking biomarker is much more applicable in metabolically active organs.

However, the reliance on a single exogenous radiotracer inherently limits its ability to precisely reflect endogenous zinc pool concentrations and spatial heterogeneity. This was illustrated by the absence of statistically significant differences in SUVs across intracerebral regions using ^63^Zn PET, suggesting a homogeneous brain uptake profile. Another significant limitation of using ^63^Zn is its short half-life of 38.5 min, which severely restricts the ability to accurately quantify slower intracellular zinc metabolism. Additionally, the spatial resolution achievable with ^63^Zn imaging is notably lower than that of ^18^F PET, a point further demonstrated in Figure 3b­(2).^107^

Regarding in vivo ^62^Zn PET imaging studies, Firth et al.’s investigation^110^ in 2023 into the effect of SLC30A8 gene loss-of-function variants (encoding ZnT8, linked to type 2 diabetes risk) on pancreatic zinc distribution, using ^62^Zn PET imaging in combination with laser ablation inductively coupled plasma mass spectrometry (LA-ICP-MS), yielded contrasting results. ^62^Zn PET imaging revealed only subtle variations in zinc trafficking across genotypes, whereas LA-ICP-MS demonstrated significant changes in endogenous zinc concentrations. This divergence indicates that ZnT8 does not play a critical role in acute ^62^Zn uptake by the pancreas, suggesting a longer-term effect on zinc accumulation. These findings strengthen the argument that radioactive zinc PET might not be a reliable indicator of the actual endogenous zinc pool in living organisms.

Thus, the development of novel human-compatible zinc-specific biosensors for nuclear imaging remains a critical unmet medical need for the precise quantification of zinc homeostasis alterations in concentration and spatial distribution. Particularly, the spatial resolution of current nuclear imaging modalities remains orders of magnitude lower than that of optical imaging, posing a major challenge for designing zinc-specific biosensors capable of reporting subtle, compartment-level changes in zinc homeostasis.

### 3.2 Radiolabeling Zinc-Specific Binding Molecules for Positron Emission Tomography Imaging of Zinc Homeostasis In Vivo

Unlike radioactive zinc ions, which enter endogenous metabolic pathways and reflect natural biodistribution and trafficking, zinc-specific binding radionuclide sensors are designed to prioritize accessibility, specificity, and stable binding to the labile zinc pool, which represents only a small fraction of total cellular zinc (∼5%) and is loosely associated with low–molecular weight ligands and MTs. In contrast, most of the zinc content (∼95%), known as immobile zinc, is tightly bound to metalloproteins and metalloenzymes, where it serves as a structural element or catalytic cofactor. Because labile zinc is more dynamic and exchangeable, it is often a more sensitive indicator of biological signaling and disease-associated changes in zinc homeostasis.^13^ In addition to intracellular pools, extracellular labile zinc also plays important physiological and pathological roles. Although extracellular zinc concentrations are typically low under basal conditions, transient increases can occur during processes such as synaptic transmission, insulin secretion, tissue injury, and inflammation. This extracellular pool may initially interact with zinc-binding radionuclide sensors and influence their distribution or apparent signal. In terms of generating imaging contrast for zinc detection, appropriate probe design—particularly with respect to membrane permeability, binding affinity, and kinetic stability—can promote intracellular trapping and retention, allowing the signal to predominantly reflect biologically relevant zinc fluxes.

Inspired by the design of fluorescent zinc biosensors that tether fluorophores to metal-ion chelators for in vivo metal sensing and visualization under microscopy,^111^ researchers have radiolabeled metal-chelating scaffolds to develop novel PET imaging radiotracers for transition metal ions. This approach was applied to test the “metal hypothesis of Alzheimer’s Disease” by Vasdev et al.,^112^ who sought to nonspecifically image the collective effects of primary in vivo metals, such as zinc, copper, and iron, in AD. To test this hypothesis, the team synthesized an ^18^F labeled metal ion chelator 2-fluoro-8-hydroxyquinoline, **1** ([^18^F]­CABS13) (Figure 5), and evaluated its potential for detecting Aβ plaques in transgenic mouse models of Alzheimer’s disease (APP/PS1) using dynamic PET imaging. The outcomes demonstrated that **1** ([^18^F]­CABS13) exhibits a washout time of less than 1 min in the normal mice brains, which is faster than the 3.5–4.5 min observed in the brains of the AD mice, indicative of a binding effect to the plaques, which slowed the washout rate in the disease mice (Figure 6a­(1–3)).

**Figure 5:**
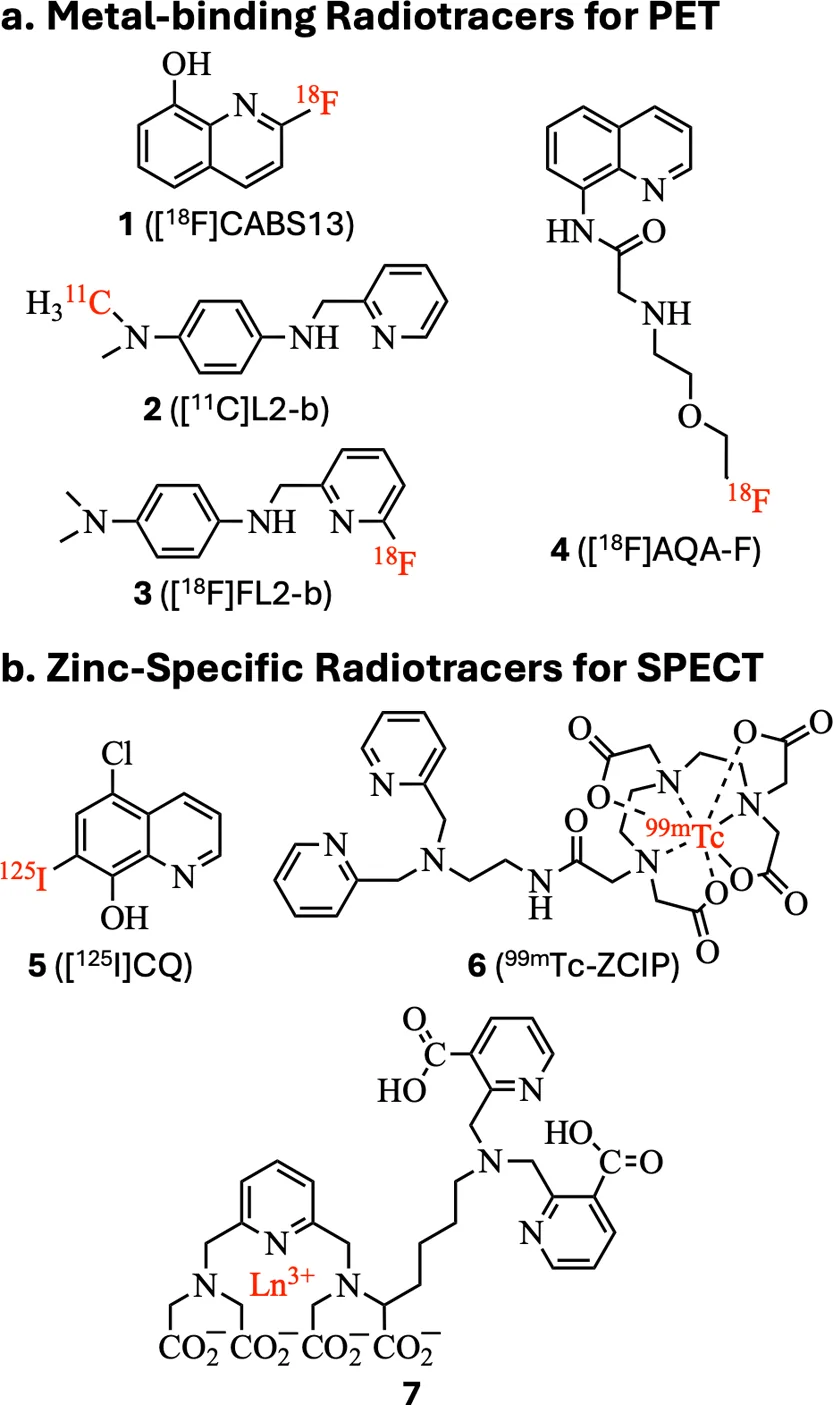
Chemical structures of zinc-specific binding radiotracers designed for (A) PET and (B) SPECT/MRI imaging of zinc homeostasis in vivo, Ln^3+^ = Gd^3+^ for MRI and ^165^Er^3+^ for SPECT, respectively.

**Figure 6:**
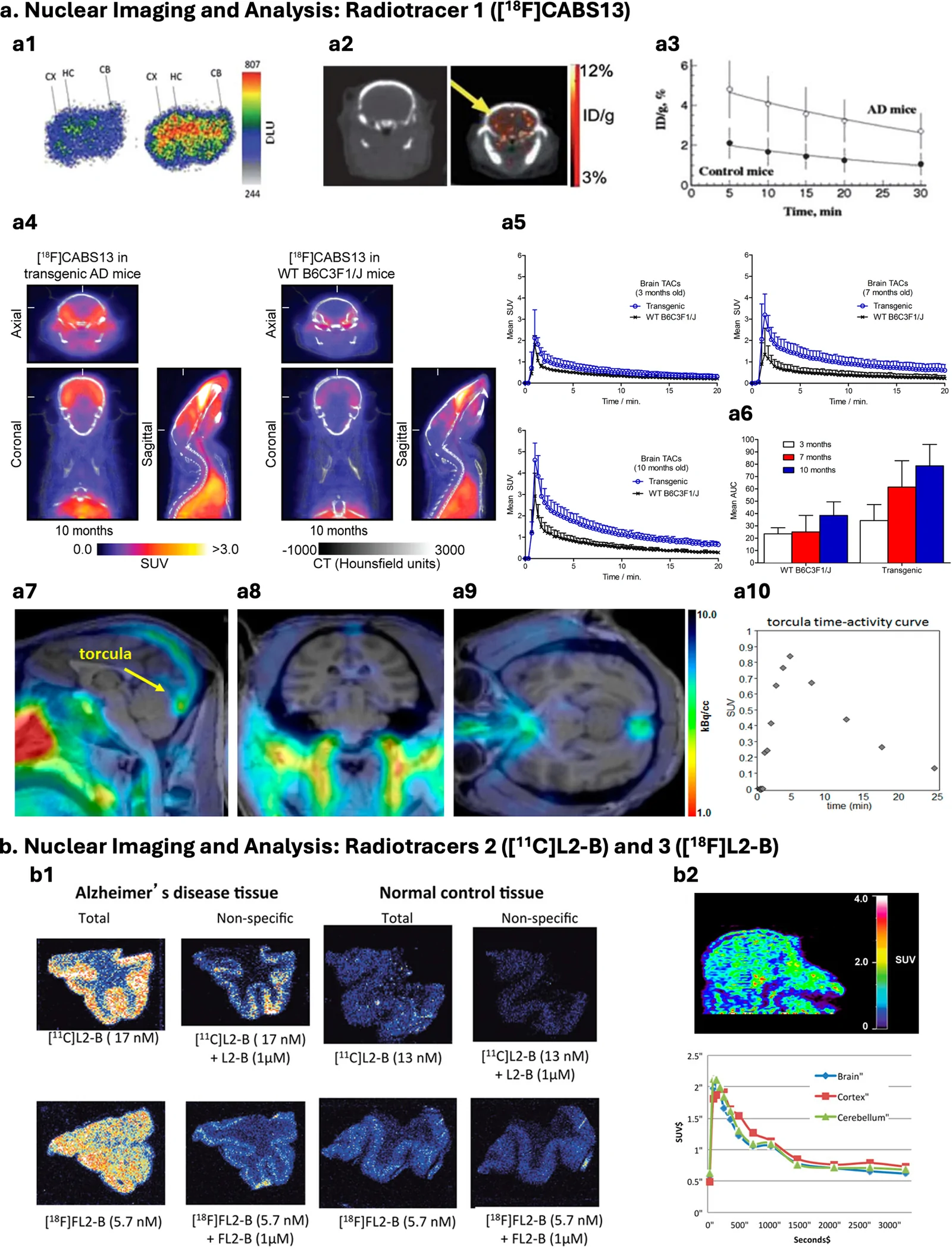
Nuclear imaging studies using three zinc-specific PET radiotracers, **1** ([^18^F]­CABS13), **2** ([^11^C]­L2-b), and **3** ([^18^F]­FL2-b) demonstrate their specific binding profiles and enhanced accumulation in Alzheimer’s disease (AD) models compared to healthy controls. (a) Imaging studies of **1** reveals its dynamic profile and specificity in AD transgenic mice models: (a(1)) Autoradiography and phosphor imaging in wild-type (left) and transgenic AD mice (right, APP/PS1, 10–12 months old) brain slices identified high zinc contents in the cortex (CX), hippocampus (HC), and cerebellum (CB), (a(2)) Coronal fused PET-CT images at 4 min postinjection illustrate rapid clearance in control mice (left) and tracer retention in the AD mouse brain (right), (a(3)) Comparative time–activity curves illustrate the difference in clearance kinetics between control and AD mice.^112^ Reproduced with permission from ref 112. Copyright 2012, Royal Society of Chemistry. (a(4–6)) A longitudinal PET comparison of **1** uptake between AD transgenic (APP/PS1) and wild-type (WT, B6C3F1/J) control mice illustrates a gradual increase in tracer accumulation in the brains of transgenic AD mice compared to wild-type (WT) controls: (a(4)) Summed axial, coronal, and sagittal PET images (5–10 min postinjection) in 10 month-old transgenic AD (left) and WT control (right) mice, (a(5)) Time–activity curves demonstrate differences in uptake over time, and (a(6)) Whole-brain AUC analysis compares tracer uptake in transgenic AD versus WT control mice at 3, 7, and 10 months, (a(7–10)) Summed PET (0–15 min) coregistered with structural MR images in a nonhuman primate: (a­(7,8)) show primary radioactivity accumulation in the confluence of the sinuses (torcula), with the occipital protuberance exhibiting the highest intensity (a­(9,10)) A time–activity curve illustrates the kinetics of the observed radioactivity.^113^ Reproduced with permission from ref 113. Copyright 2015, American Chemical Society. (b) Autoradiography and PET imaging studies of **2** and **3** demonstrate binding to AD-positive human brain tissue and characterize tracer dynamics in a nonhuman primate: (b(1)) Autoradiography images show binding of tracers to post-mortem human brain tissue from both AD-positive and normal control individuals, and (b(2)) Summed **2** PET imaging of nonhuman primate (0–60 min i.v. postinjection) (top) and corresponding time–radioactivity curves characterize tracers’ kinetics. (bottom).^116^ Reproduced with permission from ref 116. Copyright 2014, American Chemical Society.

Furthermore, the team successfully established the automated radiosynthetic method for efficiently producing **1** ([^18^F]­CABS13) with 19 ± 5% uncorrected radiochemical yield, relative to starting [^18^F]­fluoride, with ≥95% chemical and radiochemical purities, and high specific activity (>2.5 Ci/μmol) within 80 min.^113^ This advancement enabled PET/CT imaging of double mutant transgenic mice (APP/PS1) compared to age-matched wild-type mice controls (B6C3F1/J), and then performed PET/MRI imaging in nonhuman primates (*Papio Anubis baboons*). The PET images, averaged from 5–10 min postinjection of **1** ([^18^F]­CABS13), revealed an increased uptake and retention of the probe in the whole brains of the AD mice compared to the wild-type mice (Figure 6a­(4–6)). Furthermore, the older mice in both groups exhibited higher brain penetration of the probe. Measured in terms of brain peak uptake SUV, the AD mice demonstrated higher values of 2.2, 3.2, and 4.6 compared to 1.8, 1.4, and 2.9 of the wild-type mice at the ages of 3, 7, and 10 months, respectively. These promises in rodents encouraged the team to proceed to PET/MRI studies in normal baboons. The data analysis shows that the probe reaches the torcular Herophili and then distributes to the superior sagittal, occipital, and transverse sinuses (Figure 6, a(7–10)).^114^ However, only a very small proportion of the activity appears in the brain, with the possibility of merely a blood pool signal rather than blood–brain barrier (BBB) penetration. Unfortunately, the outcomes of rapid metabolism and low brain penetration in primates eventually discouraged the clinical translation of **1** ([^18^F]­CABS13).

Similarly, Carry et al. radiolabeled L2-b, a compound known for its BBB permeability and dual binding abilities with high binding affinity to Aβ aggregates and chelation to metals,^115^ with ^11^C and ^18^F isotopes to produce radiotracers **2** ([^11^C]­L2-b) and **3** ([^18^F]­FL2-b) (Figure 5), respectively, for investigating AD status.^116^ The team first used autoradiography to assess the affinity of both radiotracers in the postmortem human brain tissue from the AD-positive and normal control individuals (Figure 6b­(1)), yielding *K*_d_ values of 3.5 nM for **2** ([^11^C]­L2-b) and 9.4 nM for **3** ([^18^F]­FL2-b). The lower affinity of **3** ([^18^F]­FL2-b) is likely ascribed to the strong electron-withdrawing properties of fluorine, which reduces the electron density at the *N*,*N* metal-chelating site. Additionally, they validated the dual-binding function of the radiotracers through displacement assays using nonradioactive probes, including control molecules such as AV-45 and PiB that bind to Aβ aggregates but do not chelate metals, as well as L2-b. Encouraged by the promising results, the researchers proceeded to the PET imaging studies of **2** ([^11^C]­L2-b) in healthy nonhuman primates and confirmed its BBB permeability. Without amyloid burden, the radiotracer’s intensity reached a peak SUV = 2.0 at 2.5 min and decreased rapidly (Figure 6b­(2)).

So far, these radiolabeled sensors collectively target a range of transition metals, while zinc-specific PET imaging methods have rarely been reported and evaluated in vivo. Price et al. developed a fluorinated fluorescent probe, AQA-F, with a high-affinity binding constant (*k*_d_ = 15.2 μM) for zinc. Upon binding with zinc ions, the AQA-F undergoes an 80 nm red-shift in its emission profile from 420 nm, enabling zinc detection through fluorescence microscopy, as demonstrated in prostate and prostate cancer cell lines. Building on this structure, the team radiosynthesized ^18^F-labeled AQA-F, **4** ([^18^F]­AQA-F), for PET imaging of zinc (Figure 5).^117^ Although the team has not conducted radioactivity related studies, this strategy exhibits promise as a viable approach to designing novel radiotracers for targeting and imaging endogenous zinc in vivo.

The development of effective zinc-specific radiotracers remains a key challenge. For example, as the authors of this article, we have been actively developing zinc-chelating biosensors labeled with either ^18^F or ^13^C isotopes for using ^18^F zinc biosensor PET imaging and hyperpolarized carbon-13 MRS techniques, respectively, to image altered zinc homeostasis associated with diseases. We incorporated ^18^F directly into the pyridyl ring of the tris­(2-pyridylmethyl)­amine (TPA) scaffold, which exhibits excellent zinc-specific affinity, good membrane permeability, low cytotoxicity, and the capacity for intracellular zinc binding.^118^ We have successfully synthesized the desired ^18^F-TPA and conducted in vitro and in vivo evaluations.^119^ Unfortunately, during an in vivo PET imaging experiment with wild-type mice, we observed defluorination of the probe, indicating a recurring issue of metabolic instability when labeling fluorine to the para-position of an amine on an aromatic ring, such as pyridine or aniline.^120^ Subsequently, we optimized the probe structures by conjugating the TPA scaffold to an ^18^F-benzamide moiety via a diamine linker. By modulating the linker composition, we successfully tuned the lipophilicity of the probes to optimize their pharmacokinetics for in vivo PET studies.

### 3.3 Radiolabeling Zinc-Specific Binding Molecules for Single-Photon Emission Computed Tomography Imaging of Zinc Homeostasis In Vivo

The development of zinc-specific radiotracers for SPECT is another indispensable direction for in vivo zinc imaging. Similar to PET zinc biosensors, these initial SPECT sensors incorporated radioactive moieties and metal-specific chelators to investigate the metal hypothesis of AD. For example, Papazian and Opazo et al. radio-iodinated clioquinol (CQ) to produce **5** ([^125^I]­CQ) (Figure 5) for a pilot SPECT study of imaging zinc in AD models and human AD patients.^121,122^ CQ is a well-known zinc and copper chelator in clinical trials. It crosses the BBB and inhibits the precipitation of Aβ aggregates by copper and zinc ions, thereby preventing cognitive deterioration.^123^ The APP transgenic (Tg2576) mice injected with **5** ([^125^I]­CQ) showed increased retention of the probe in the brain compared to the non-Tg mice, and subsequent autoradiography of brain sections confirmed the specific **5** ([^125^I]­CQ) enrichment in the neocortex (Figure 7a­(1)). However, in AD patients injected with **5** ([^125^I]­CQ) for SPECT imaging, the signal was inadequate for proper imaging, likely due to the low retention of the probe (Figure 7a­(2)). This suggests a need for essential optimization, such as through improved formulation or delivery approaches.

**Figure 7:**
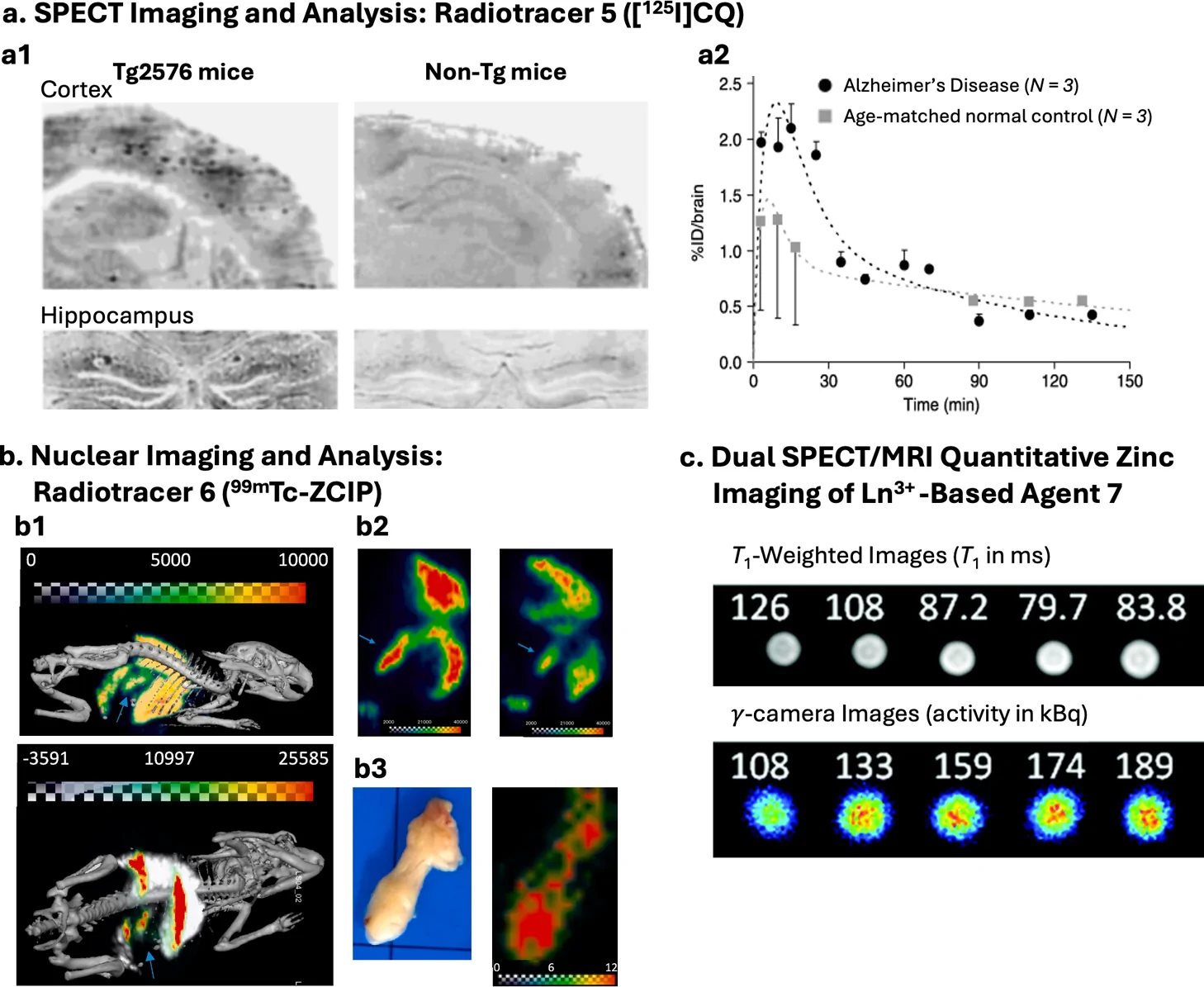
SPECT in vivo and phantom nuclear images demonstrate the feasibility of using zinc-specific SPECT radiotracers, **5** ([^125^I]­CQ), **6** (^99m^Tc-ZCIP), and **7** (^165^Er­(III) and Gd­(III)) to image elevated zinc levels in murine models and quantify them in phantoms. (a) In vivo imaging with **5** showed that the tracer can distinguish between subjects with mild AD and healthy controls by its retention kinetics, consistent with elevated zinc levels in AD pathology: (a(1)) Coronal film-emulsion autoradiography images show binding in the cortex and hippocampus of both Tg2576 and non-Tg mice, (a(2)) SPECT-measured retention of **5** over time in the brains of subjects with mild AD (*N* = 3) or healthy aging controls (AC, *N* = 3) illustrates enhanced binding and retention in the brains of subjects with mild AD compared to controls.^122^ Reproduced with permission from ref 122. Copyright 2006, John Wiley & Sons Ltd. and The Anatomical Society. (b) in vivo and ex vivo nuclear imaging with **6** confirmed its specific pancreatic uptake in mice: (b­(1,2)) in vivo SPECT images 30 min postinjection show pancreatic uptake (indicated by arrows), which is blocked by preinjection of cold tracer, and (b(3)) The specificity of the tracer is further confirmed by an ex vivo gamma camera image (right) of the extracted pancreas (left).^124^ Reproduced with permission from ref 124. Copyright 2021, Elsevier Ltd. (c) A dual SPECT/MRI phantom study using ^165^Er­(III) and Gd­(III) cocktail **7** demonstrate the possibility of quantitative zinc imaging: *T*_1_-weighted MRI images (top) of five samples were acquired at 1.5 T using a spin–echo sequence (TE = 9, TR = 100 ms) and *T*_1_ values (ms) were measured with variable TR from 20 to 2000 ms. Corresponding gamma camera image (bottom) shows the activities in kBq.^127^ Reproduced with permission from ref 127 Copyright 2018, Royal Society of Chemistry.

To enable quantitative SPECT imaging of zinc in the pancreas, Eeda et al. introduced the most practical radiotracer to date: the ^99^mTc-labeled zinc-chelating imaging agent **6** (^99m^Tc-ZCIP), which couples an imaging moiety of ^99m^TC coordinated diethylene triamine pentaacetic acid (DTPA) to a zinc-specific chelating group—bispicolyethylamine (BPEN) (Figure 5).^124^ The advantages of this method are 2-fold: first, BPEN exhibits a strong binding affinity for zinc, as demonstrated by its ability to displace zinc from dithizone coordination—a zinc-specific binding indicator commonly used for staining and counting pancreatic islet β cells.^125^ Second, ^99m^Tc has an optimal γ-ray of 140 keV and a half-life of 6 h, providing excellent imaging quality with a low radiation burden. Additionally, ^99m^Tc is readily accessible, enhancing its widespread applicability in preclinical and clinical research. The team administered ∼0.3 mCi of **6** (^99m^Tc-ZCIP) to CD-1 mice for SPECT imaging, observing a highly resolved image of the pancreas in the midposterior region, which is consistent with the high pancreatic uptake of **6** (^99m^Tc-ZCIP) observed during postnecropsy ex vivo autoradiography imaging of the pancreas (Figure 7b). These findings suggest the potential feasibility of using **6** (^99m^Tc-ZCIP) to detect and stage pancreatic diseases. However, the current report has not thoroughly explored the quantitative detection of zinc.

Current advances in clinically translatable in vivo zinc imaging methods remain largely qualitative rather than quantitative, a limitation that hinders the full potential for precision medicine. For example, zinc deficiency is a reliable biomarker for prostate cancer, where zinc concentration could decrease by as much as 80%.^10–12^ Similarly, zinc quantification can facilitate the diagnosis of exocrine pancreatic dysfunction with a sensitivity and a specificity of 97% and 91%, respectively.^126^ Nuclear imaging techniques such as PET and SPECT, similar to optical methods, have the potential to reflect the endogenous zinc concentrations, facilitating the quantitative mapping of its spatial and temporal distribution in various physiological and pathological contexts. This approach can not only monitor zinc as a reliable biomarker for precision medicine, but also bridge the knowledge gap between local cellular dysfunction and systemic diseases. However, due to significant challenges and limitations, only a few nuclear imaging sensors, such as **4** ([^18^F]­AQA-F) and **6** (^99m^Tc-ZCIP), have been reported.

To address this limitation, Malikidogo et al. developed a dual-modality cocktail of SPECT and MRI zinc sensor **7**, consisting of a bis­(pyridinylmethyl)­amine scaffold substituted with two carboxylate groups for zinc targeting, linked to a moiety containing two aminocarboxylate substituted pyridine for chelating the imaging contrast lanthanide ions (Ln^3+^, such as Gd^3+^ or ^165^Er^3+^).^127^ The team utilized a cocktail consisting of two distinct complexes: the zinc-responsive MRI contrast agent Gd-7 (Gd^3+^-chelated) and its radioactive SPECT analog ^165^Er-7 (^165^Er^3+^-chelated, *t*_1/2_ = 10.36 h, electron capture with 50 keV X-ray emissions^128^). While Gd-7 exhibits increased relaxivity upon ternary binding of zinc ions and human serum albumin (mechanism discussed in the following section), the concentration-independent SPECT signal from ^165^Er-7 serves as a radiometric internal standard.

Based on the relative sensitivities of MRI and SPECT detection, the researchers combined these complexes at a molar ratio of 1:2.4 × 10^–7^ (Gd/Er). They serially diluted this mixture to produce five samples, each of which was added with an unknown amount of zinc in the presence of HSA (0.6 mM). As shown in Figure 7c, for each sample, the ^165^Er concentration was determined by its signal intensity on the γ-camera; this value was then used to calculate the corresponding Gd^3+^ concentration using the known mixing ratio. After measuring the longitudinal relaxation time (*T*_1_), the relaxivity (*r*_1_, mM^–1^ s^–1^), defined as the ability of the probe to increase the water proton relaxation rate (1/*T*_1_) per unit of its concentration (mM), was determined. Finally, by using a calibration curve of *r*_1_ versus the [Zn^2+^]/[Gd-7] ratio, the concentration of zinc ions in the samples was accurately quantified. While dual-modality zinc sensing is still in its infancy and lacks in vivo validation, this work provides a successful proof-of-concept in phantom studies. By combining the high sensitivity of SPECT with the superior spatial resolution of MRI, the researchers demonstrated the potential for accurate, quantitative zinc assessment.

## 4 Magnetic Resonance Imaging for Imaging Free Zinc In Vivo

### 4.1 Zinc-Responsive Relaxivity MRI Contrast Agents

Clinical MRI predominantly images proton signals from free water to construct the anatomical structures of human organs. By leveraging different proton nuclear spin relaxation rates in various microenvironments with specifically designed pulse sequences and parameters, MRI provides essential soft-tissue imaging contrast for detecting and assessing diseases such as tumors. In many situations, a contrast-enhancing agent, such as a paramagnetic metal ion complex (e.g., Gd^3+^, Mn^3+^, and others), is administered to enhance the image contrast and improve diagnostic accuracy. This enhancement is attributed to the interactions between the unpaired electrons of paramagnetic metal ion and the water molecules bound in its inner sphere, and the rapid exchange of these inner-sphere-bound water molecules with the bulk solvent water molecules.^129^ The efficiency of an MRI contrast agent depends on its ability to modulate the longitudinal relaxation rate of water protons (1/T_1_) per unit concentration—the relaxivity value (*r*_1_, mM^–1^ s^–1^) as defined above. According to the Solomon-Bloembergen-Morgan theory, *r*_1_ is influenced by multiple physicochemical parameters, including electronic relaxation times, interatomic distances, and zero-field splitting constants. In practice, relaxivity can be most effectively optimized through structural modulation of three key “tunable” parameters: the hydration number (*q*), defined as the number of water molecules directly coordinated to the paramagnetic center; the rotational correlation time (τ_R_) of the complex, which reflects the molecular tumbling rate of the complex; and the mean residence lifetime of the inner-sphere water molecules (τ_m_), which described how long a coordinated water molecule remains bound before exchanging with bulk water. It is the inverse of the water exchange rate (*k*_ex_), where τ_m_ = 1/*k*_ex_ (Figure 8a).^130,131^ Accordingly, the rational design of zinc-responsive MRI contrast agents relies on triggering detectable changes in these specific parameters in response to zinc binding to enable MRI imaging in both in vitro and in vivo settings.

**Figure 8:**
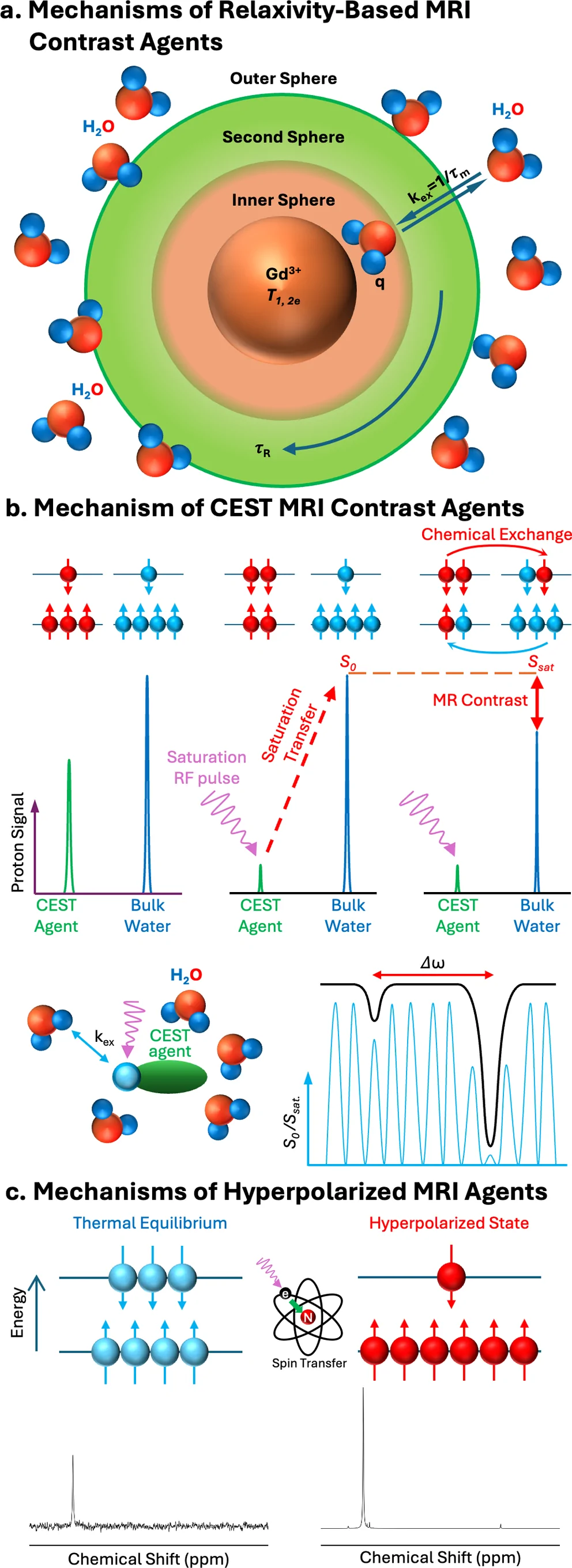
Three primary MRI mechanisms for designing zinc-responsive contrast agents, including (a) paramagnetic metal ion-based relaxivity, (b) chemical exchange saturation transfer, and (c) nuclear hyperpolarization.

In 2001 and 2002, the Nagano laboratory designed the first zinc-responsive relaxivity MRI contrast agent based on changes in the hydration number (*q*) in response to zinc binding.^132,133^ The zinc contrast agent, such as contrast agent **8** in Figure 9, consists of two types of metal chelators: diethylenetriaminepentaacetic acid (DTPA) for coordinating Gd^3+^ and bisamide zinc-specific chelating groups, such as *N*,*N*-(2-pyridyl-methyl)­(carboxy-methyl)­ethylene diamine, tethered at each side of DTPA for zinc ion binding. This zinc chelating structure exhibits significant sensitivity and excellent specificity for zinc over other metal ions, such as calcium and magnesium. Upon zinc binding, the molecule undergoes a tight close-up conformational change that restricts water access to the Gd^3+^ center. As a result, the researchers observed a zinc concentration-dependent (0–1 equiv relative to contrast agent **8**) decrease in *r*_1_ from 4.8 to 3.4 mM^–1^ s^–1^ at 60 MHz and 37 °C, attributed to a reduction in the hydration number (*q*). In the subsequent MRI study, mixing contrast agent **8** with 1 equiv of zinc in water produced a bright-to-dark (negative) change in *T*_1_-weighted signal intensity. As expected, the probe also demonstrated strong selectivity for zinc ions over competing metal ions, such as calcium and magnesium ions. However, this response generates a negative contrast, in which zinc binding reduces MRI signal due to a lower *r*_1_ value. Such negative contrast is considered less desirable for image detection, as the signal loss can be confounded with other physiological or technical sources of MRI signal attenuation.

**Figure 9:**
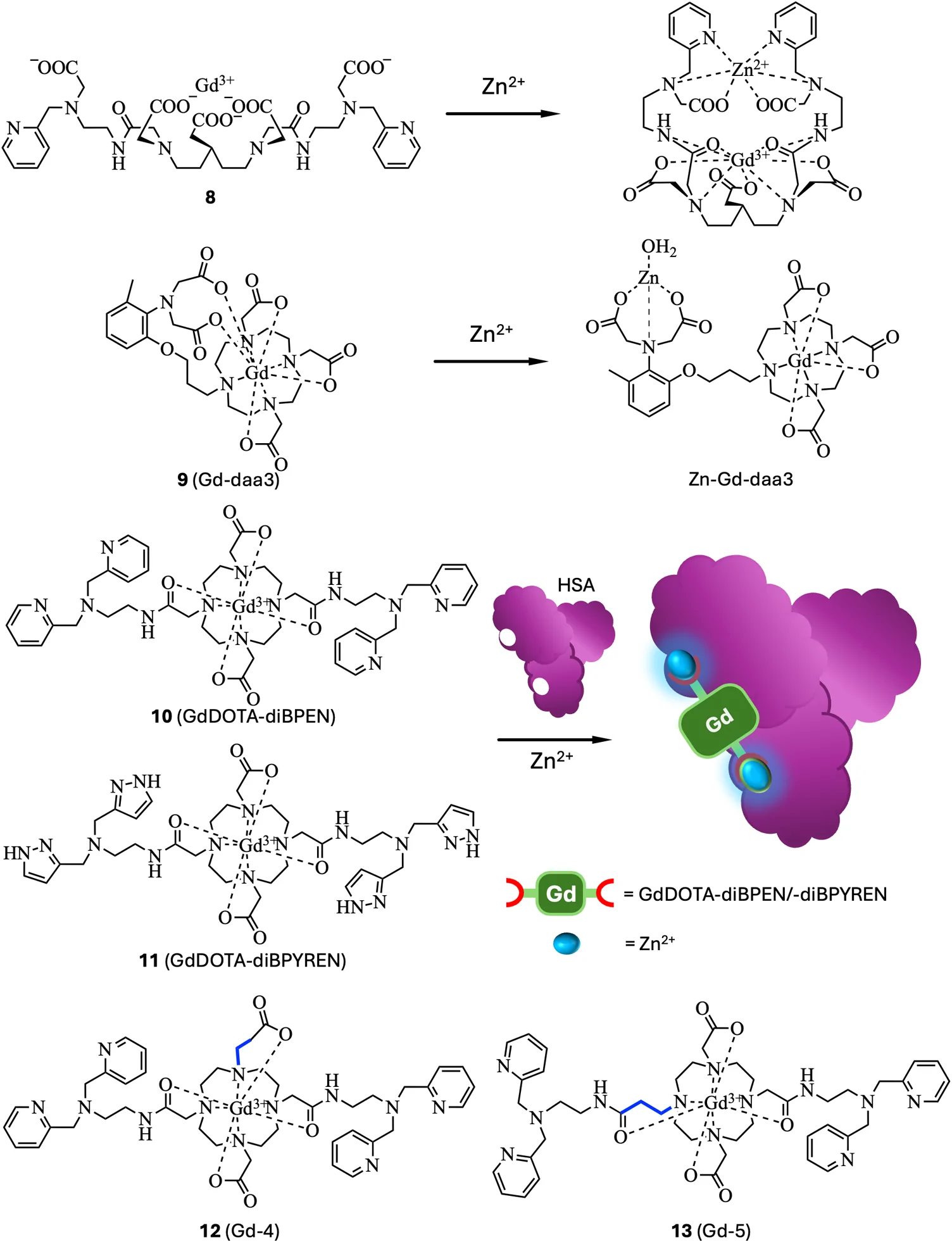
Chemical structures of relaxivity-based zinc-responsive MRI contrast agents and proposed mechanisms of their capability of zinc detection.

Therefore, to brighten the MRI contrast upon zinc binding, an opposite molecular conformational change, which allows water access to the Gd^3+^ center, is required. Major et al. first exploited this strategy by synthesizing the contrast agent **9** (Gd-daa3) in Figure 9, which consists of a paramagnetic center (GdDO3A) and an aminoacetate-based zinc-binding moiety.^134,135^ In the absence of zinc, the carboxylate groups of the aminoacetate moiety coordinate to the Gd^3+^ center, restricting water access and minimizing the *r*_1_ value. Upon zinc binding, these groups detach from the GdDO3A center, allowing the entry of a water molecule into the inner coordination sphere. As a result, *r*_1_ increases from 2.33 to 5.07 mM^–1^ s^–1^ (37 °C, pH 7.4, 60 MHz), consistent with a change in the hydration state to *q* = 1. This conformational change generates a positive contrast as preferred for MRI contrast agents and enables contrast agent **9** (Gd-daa3) to achieve an in vitro zinc detection limit as low as 100 μM. Although the researchers did not conduct in vivo evaluations, they estimated the performance of contrast agent **9** (Gd-daa3) by comparing it to the standard Gadolinium diethylenetriaminepentaacetate (Gd-DTPA, 3.8 mM^–1^ s^–1^ at 37 °C and 60 MHz). Relative to the standard, contrast agent **9** (Gd-daa3) exhibits a 40% lower relaxivity before zinc binding and a 33% higher relaxivity afterward; this significant shift indicates sufficient sensitivity for in vivo detection.

In addition to modulating the hydration number (*q*), researchers have investigated strategies to reduce the molecular tumbling rate upon zinc binding, thus increasing the rotational correlation time (τ_R_), which enhances relaxivity (*r*_1_) and improves zinc detection sensitivity. For instance, Esqueda et al. designed a new Gd^3+^-based MRI zinc sensor **10** (GdDOTA-diBPEN) (Figure 9).^136^ The binding affinity of this agent for human serum albumin (HSA) increases significantly upon forming a ternary complex with two equivalents of zinc, leading to slower molecular tumbling and a consequently longer rotational correlation time (τ_R_). This mechanism results in at least a 3-fold increase in relaxivity (*r*_1_), from 6.6 ± 0.1 to 17.4 ± 0.5 mM^–1^ s^–1^ in the Tris buffer system (0.1M, pH 7.6) in the presence of 0.6 mM HSA at 37 °C and 23 MHz, thereby enhancing zinc detection sensitivity to 30 μM. This high-relaxivity zinc-responsive MRI contrast enabled in vivo MRI imaging of zinc release from pancreatic β-cells during glucose-stimulated zinc secretion (GSZS) in mice (Figure 10a­(1–3)),^137^ and detected differential zinc release from healthy versus malignant mouse prostates (Figure 10a­(4)).^138^

**Figure 10:**
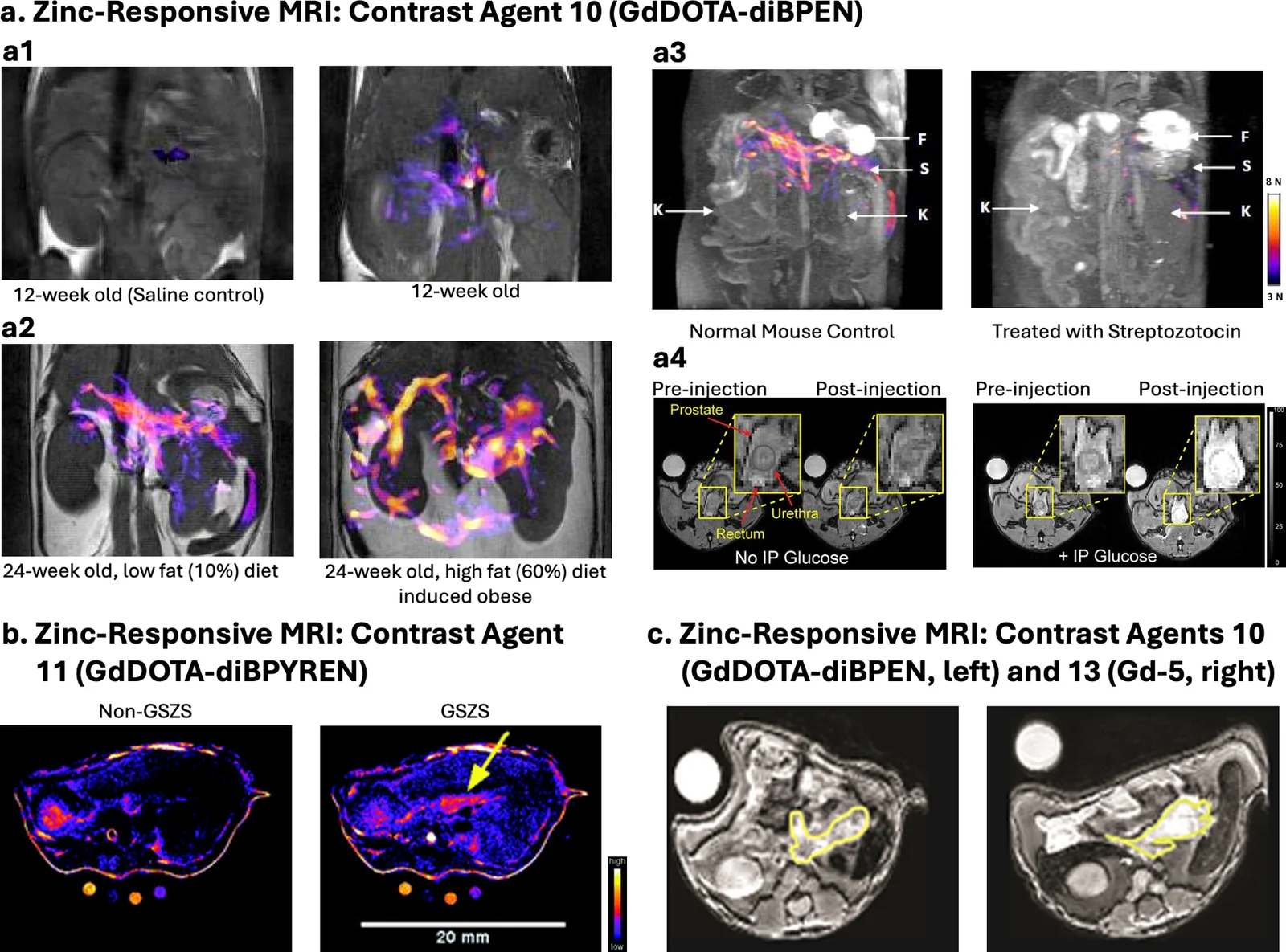
MRI images acquired using relaxivity-based zinc-responsive contrast agents, **10** (GdDOTA-diBPEN) and **11** (GdDOTA-diBPYREN), demonstrate the feasibility of imaging glucose-stimulated zinc secretion (GSZS) in mouse models and correlating with the status of the models. (a) MRI images acquired with **10** showing GSZS-related contrast enhancements across various pancreatic conditions: (a(1)) A comparison between saline-injected and glucose-injected mice shows that glucose injection resulted in a strong GSZS-related contrast enhancement, (a(2)) A comparison between mice on a standard fat diet versus a high-fat diet shows that the high-fat diet model resulted in a higher GSZS-related contrast enhancement, (a(3)) A comparison between a control mouse and a streptozotocin-induced diabetes model demonstrates a loss of GSZS enhancement in the diabetes model.^137^ Reproduced with permission from ref 137. Copyright 2011, The National Academy of Sciences of the USA. and (a(4)) The contrast enhancement is also demonstrated in the mouse prostate, showing a significant GSZS enhancement in the presence of glucose injection.^138^ Reproduced with permission from ref 137. Copyright 2016, The National Academy of Sciences of the USA. (b) MRI images acquired with **11** showing GSZS enhancements.^139^ Reproduced with permission from ref 139. Copyright 2012, Royal Society of Chemistry and (c) A direct comparison of 10 and **13** (Gd-5, right) demonstrates **13** provided a more significant enhancement for GSZS: the mouse pancreas (yellow outline) showed a significant contrast enhancement with **13** (right) compared with **10** (left).^141^ Reproduced with permission from ref 141. Copyright 2015, American Chemical Society.

Subsequently, the researchers substituted the BPEN zinc-chelating groups with *N*,*N*-bis­(3-pyrazolyl-methyl) ethylene diamine groups (BPYREN) to create the second-generation zinc-responsive contrast agent **11** (GdDOTA-diBPYREN) (Figure 9). As expected, the contrast agent exhibits suboptimal binding affinity for HSA by it alone, but it undergoes a remarkable increase in relaxivity (*r*_1_) upon binding with zinc, rising from 8.4 ± 0.2 to 15.3 ± 0.4 mM^–1^ s^–1^ at 37 °C and 23 MHz in the Tris buffer system (0.1 M, pH 7.6) in the presence of 0.6 mM HSA.^139^ Importantly, under physiological conditions in human blood serum, contrast agent **11** (GdDOTA-diBPYREN) exhibits an increase in relaxivity (*r*_1_) from 6.0 ± 0.1 to 13.1 ± 0.1 mM^–1^ s^–1^, which is three times greater than the increase observed with **10** (GdDOTA-diBPEN) from 6.1 to 8.6 mM^–1^ s^–1^. A comparison of MRI images, pre- and postglucose injection, is presented in Figure 10b.

Despite these efforts to develop zinc-responsive MRI contrast agents, none have demonstrated a particularly high *r*_1_ value upon zinc binding for highly sensitive zinc detection. Sherry and Wu emphasized that the water exchange rate (*k*_ex_, where *k*_ex_ = 1/τ_*m*_) at the paramagnetic center is a critical parameter, yet it is overlooked in the design of new responsive MRI agents.^140^ Ultimately, the Sherry team synthesized a series of **10** (GdDOTA-diBPEN) derivatives, which continue to utilize HSA binding to modulate the rotational correlation time of the sensor (τ_R_), but with fine-tuned structures to investigate the water exchange rate (k_ex_) between the water coordinated to the inner sphere of Gd^3+^ and bulk solvent water. Two structures, **12** (Gd-4) and **13** (Gd-5) in Figure 9, exhibited exceptionally high relaxivity (*r*_1_) values of 47.6 ± 1.2 and 50.1 ± 1.2 mM^–1^ s^–1^ at 0.47 T and 37 °C, respectively, representing a 3-fold improvement over the parent **10** (GdDOTA-diBPEN) and enabling highly sensitive zinc-responsive MRI to detect zinc ions released from the mouse pancreas upon GSZS at a low field of 0.47 T (Figure 10c).^141^ However, the researchers did not measured the *r*_1_ changes upon zinc binding in the presence of HSA (0.6 mM) to quantify signal enhancement; nevertheless, they demonstrated that optimizing the water exchange rate is an effective strategy for developing more sensitive zinc-responsive MRI contrast agents.

Over the past decade, the Sherry laboratory has focused on developing novel zinc-responsive MRI agents to image GSZS from prostate tissue^138,142–144^ and pancreatic β-cells.^137,143,145–147^ Their innovative work spans a diverse array of zinc-specific imaging techniques, including manganese-based MRI contrast agents for GSZS detection in the mouse pancreas and prostate,^148,149^ ZIMIR imaging of oscillatory insulin secretion in mouse pancreatic islet cells,^150^ synchrotron radiation X-ray fluorescence for trace elemental mapping in prostate cancers,^151,152^ the development of paraCEST zinc sensing agent^153,154^ and hyperpolarized ^15^N tris­(2-pyridylmethyl)­amine MRI sensor for detecting mobile Zn^2+^ with more details discussed in the following section.^155^ This extensive research facilitates the assessment of the biological role of zinc and its metabolism in vivo and accelerates progress toward clinical translation. Additionally, the team has published several comprehensive review articles summarizing advancements in in vivo zinc responsive MRI through 2020.^17,19,140,156,157^

It is also crucial to highlight the significant contributions from other research teams. For instance, Bonnet et al. have focused on designing novel metal-sensing MRI methods. A notable achievement includes their fast-field cycling MRI approach, which uniquely enables zinc detection at 3 T–a feat not possible with the same contrast agent using conventional MRI approaches at this field strength.^158^ The team has been dedicated to revealing the underlying mechanisms,^159^ leading to a large body of publications. Furthermore, they have provided comprehensive reviews from various perspectives on the development of metal-sensing MRI agents and imaging methods.^160–163^

In addition to relaxivity-dependent MRI zinc imaging, researchers have incorporated fluorescent functionality into these structures to develop dual-modality imaging methods. This approach enables the covalidation of contrast agent accumulation at target sites.^164^ For example, Stasiuk et al. designed a dual-modal MRI/fluorescence zinc-responsive imaging agent **14** (Figure 11) by tethering a zinc-chelating amidoquinoline moiety to a Gd-DOTA complex center.^165^ This contrast agent exhibits a unique large ratiometric Stokes shift in the fluorescence from λ_em_ = 410 to 500 nm and an increase in relaxivity (*r*_1_) from 4.2 to 6.6 mM^–1^ s^–1^, followed by a decrease to 4.9 mM^–1^ s^–1^ (400 MHz, 298 K) upon binding to zinc. The in vitro fluorescence study demonstrated its localization in the secretory granules of single β-cells, with intensity dependent on zinc levels. In the subsequent in vivo MRI study with its control, Gd-DOTA, the results indicated cellular uptake in the endocrine pancreas of C57/BL6 mice, demonstrating its potential for assessing β-cell mass (Figure 12a).

**Figure 11:**
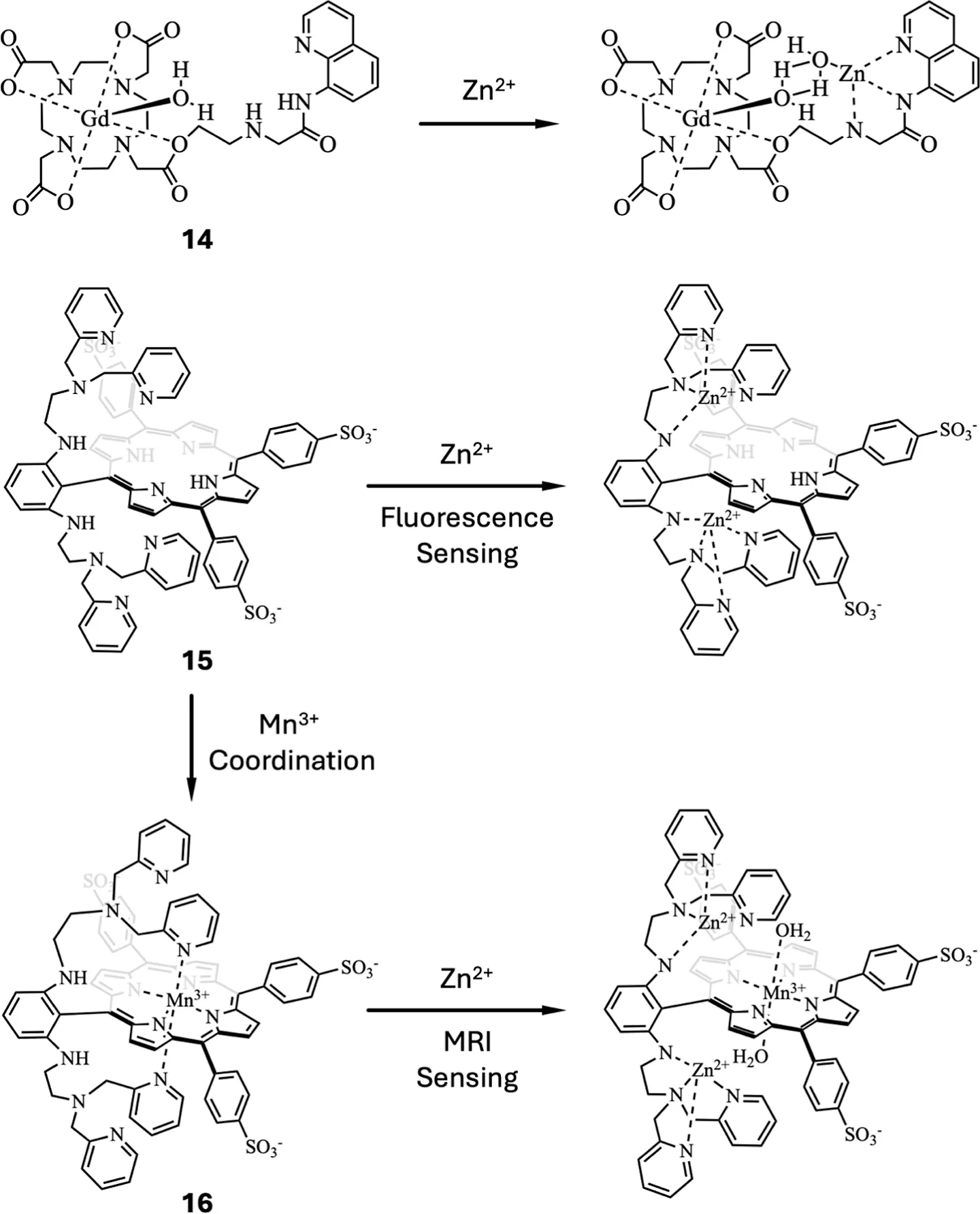
Chemical structures of dual relaxivity and fluorescence zinc-specific contrast agents.

**Figure 12:**
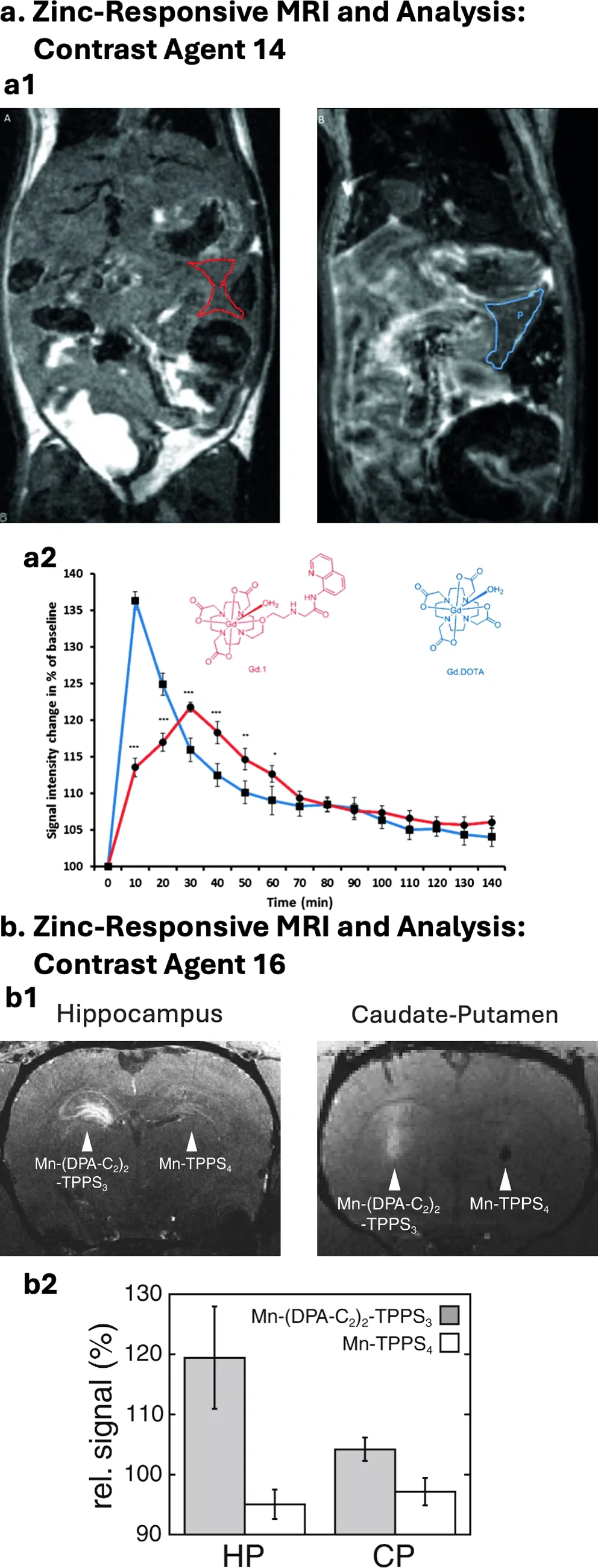
MRI images demonstrate signal enhancement with two zinc-responsive dual-mode MRI and fluorescent contrasts, **14** and **16**, compared to nonzinc-specific controls in separate studies of the murine pancreas and brain. (a) In vivo MRI images and signal enhancement kinetics show that **14** provides an overall signal enhancement in the mouse pancreas compared to the control contrast Gd·DOTA: (a(1)) MRI images of mice pancreas following injection of **14** (left) and the control contrast Gd·DOTA (right), (a(2)) A time-course plot (*n* = 10) showing the relative signal intensity change over 40 min.^165^ Reproduced with permission from ref 165. Copyright 2015, Wiley-VCH Verlag GmbH & Co. KGaA. (b) In vivo MRI images and signal quantification from rat brains confirm the zinc-dependent uptake and signal enhancement by **16** compared to the nonzinc-specific control Mn-TPPS4: (b(1)) In vivo MRI imaging from the rat brain showing the signal enhancement with **16** injected on the left side of hippocampus (HP) and Caudate-Putamen (CP), compared to the right side of each region injected with the control Mn-TPPS_4_, and (b2) A relative MRI signal quantification plot confirms the zinc-dependent uptake of **16** in these regions, highlighting its sensitivity and zinc-specificity.^167^ Reproduced with permission from ref 167. Copyright 2010, Elsevier Ltd.

In another example, Zhang et al. tethered a di-DPA group to a porphyrin scaffold to create fluorescent zinc sensor **15** ((DPA-C_2_)-TPPS_3_), which, upon insertion of Mn^3+^, converts into MRI contrast agent **16** ([(DPA-C_2_)_2_-TPPS_3_Mn­(III)]) (Figure 11).^166^ Compared to **14**, this molecule demonstrates transmembrane permeability due to its hydrophobic surface area and the charge delocalization across the aromatic ring of its metalloporphyrin scaffold, enabling the detection of intracellular zinc and offering particular value for the study of neurobiological functions and diseases. Thus, the team injected **16** ([(DPA-C_2_)_2_-TPPS_3_Mn­(III)]) and Mn-TPPS4 (a non-zinc-chelating control) into the hippocampus (zinc-rich region) and caudate-putamen (less zinc region) in the left and right sides of a rat brain, respectively.^167^ The MRI data demonstrated excellent specificity and sensitivity for detecting and differentiating subtle differences in zinc levels between these two brain regions (Figure 12). After in vivo imaging, this sensor also functioned as a histological dye in ex vivo studies, confirming the consistency of its biodistribution and concentration in the regions with enhanced MRI contrast. Given the possible environmental influence on MRI signals, analyzing the post-mortem distribution using optical methods facilitates quantitative interpretation of molecular imaging results and localization of the sensor on length scales below the resolution of MRI.

### 4.2 Zinc-Responsive CEST MRI Contrast Agents

Chemical Exchange Saturation Transfer (CEST) is an emerging MRI imaging technique that utilizes specific chemical exchange pathways between nearby molecules to image pathological changes in biomarkers, including tissue pH, ions, enzyme activity, and redox state.^168^ CEST MRI relies on the selective saturation of labile protons in contrast agents, which resonate at distinct frequencies. These saturated protons exchange with bulk water, attenuating the water MR signal and creating negative contrast (Figure 8b).^169,170^ To detect CEST signals, the sensor molecules’ protons should have a sufficiently fast exchange rate with bulk water protons, balanced by a slow relaxation rate. In other words, the frequency difference (Δω) between the two proton pools should be greater than or equal to the rate of exchange (*k*_ex_) (Δω ≥ *k*_ex_) (Figure 8b, bottom).^169,171^ Therefore, paramagnetic metals are used to increase Δω for enhancing the sensitivity of CEST imaging, known as paraCEST. Using agents without metal ions is termed diaCEST.^169,172^

#### 4.2.1 Zinc-Responsive DiaCEST MRI Contrast Agents

DiaCEST agents are usually low-molecular-weight diamagnetic molecules with exchangeable protons, such as those in amine, amide, and hydroxyl groups of sugars, amino acids, and other endogenous metabolites. They usually show good solubility, biocompatibility, and sensitivity for clinical translation.^172^ However, the main limitation of diaCEST agents is their narrow range of Δω (0.8–10 ppm), making it difficult to distinguish the spins between two proton pools because numerous other exchangeable protons are present within this range in vivo.^168,173^

To address the limitations of proton-based DiaCEST, researchers have developed ^19^F-labeled zinc-sensing molecules that exploit the CEST phenomenon on fluorine rather than protons. This ion CEST (iCEST) approach has emerged as a sensitive and background-free MRI modality for the precise detection of physiological metal ions. By leveraging the wide chemical shift range of fluorine, iCEST clearly resolves the distinct signals of “free” and “ion-bound” sensors. The mechanism involves applying a frequency-specific saturation pulse to the metal-bound complex; the resulting saturation is then transferred through chemical exchange to the free sensor pool, amplifying the signal from trace amounts of mobile zinc ions. Moreover, because each metal ion interacts with the sensor via a unique saturation exchange rate (*k*_ex_) and ^19^F chemical shifts (Δω), these parameters act as a spectroscopic “fingerprint” that enables metal-specific detection with high spatial resolution in deep tissues. This selective sensing capability has been successfully demonstrated with iCEST probes such as **17** (5F-BAPTA),^174,175^ **18** (TF-BAPTA),^176^ and others **19** and **20** ^177^ (Figure 12).

Leveraging the advantages over proton-based diaCEST, Yuan et al. conducted iCEST imaging with **18** (TF-BAPTA) to evaluate the impact on the functionality of d-glucose-stimulated zinc secretion in prostate cancer mouse models compared to normal prostate tissue.^178^ The normal prostate mice showed a dramatic increase in iCEST signal from 4.9 ± 3.4% to 21.7 ± 5.1% following glucose stimulation. This response was absent in orthotopic mouse models xenografted with LNCaP (androgen-dependent) and DU145 (androgen-independent) cells into the anterior prostate of immunodeficient NSG mice (Figure 14a­(1)).

Furthermore, the team investigated various stages of the transgenic adenocarcinoma of the mouse prostate (TRAMP) model, which histologically and biochemically resembles the human clinical progression from prostatic intraepithelial neoplasia to a dedifferentiated phenotype over time.^179,180^ The studies showed an apparent decline in iCEST signals with the age of the mice, decreasing from 20.4 ± 4.3% to 17.9 ± 3.8%, and further to 9.6 ± 3.1% at 10, 17, and 24 weeks, respectively (Figure 14a­(2)). The results of all these models were consistent with the presence of a zinc-deficient environment in poorly differentiated adenocarcinoma. However, despite its promise, **18** (TF-BAPTA) exhibits low CEST signal enhancement, significantly limiting the dynamic range of detectable ion concentrations.^181^

To achieve maximal signal amplification for wide-range detectability of zinc concentrations and spectrally resolved zinc specificity using iCEST, Tirukoti et al. investigated the kinetic and thermodynamic features of the fluorinated zinc-chelating sensors. They concluded that an optimal probe requires a fast zinc-dissociation rate (K_off_) and weak zinc-binding affinity (K_d_). They demonstrated this by synthesizing a fluorinated 2-(bis­(2-pyridylmethyl)-amino)-ethanol contrast agent **20** (Figure 13), which enabled the mapping of labile zinc distribution and differentiation between the zinc-rich Cornu Ammonis 3 (CA3) and zinc-poor thalamus regions in the hippocampus of a live rat (Figure 14b).^181^

**Figure 13:**
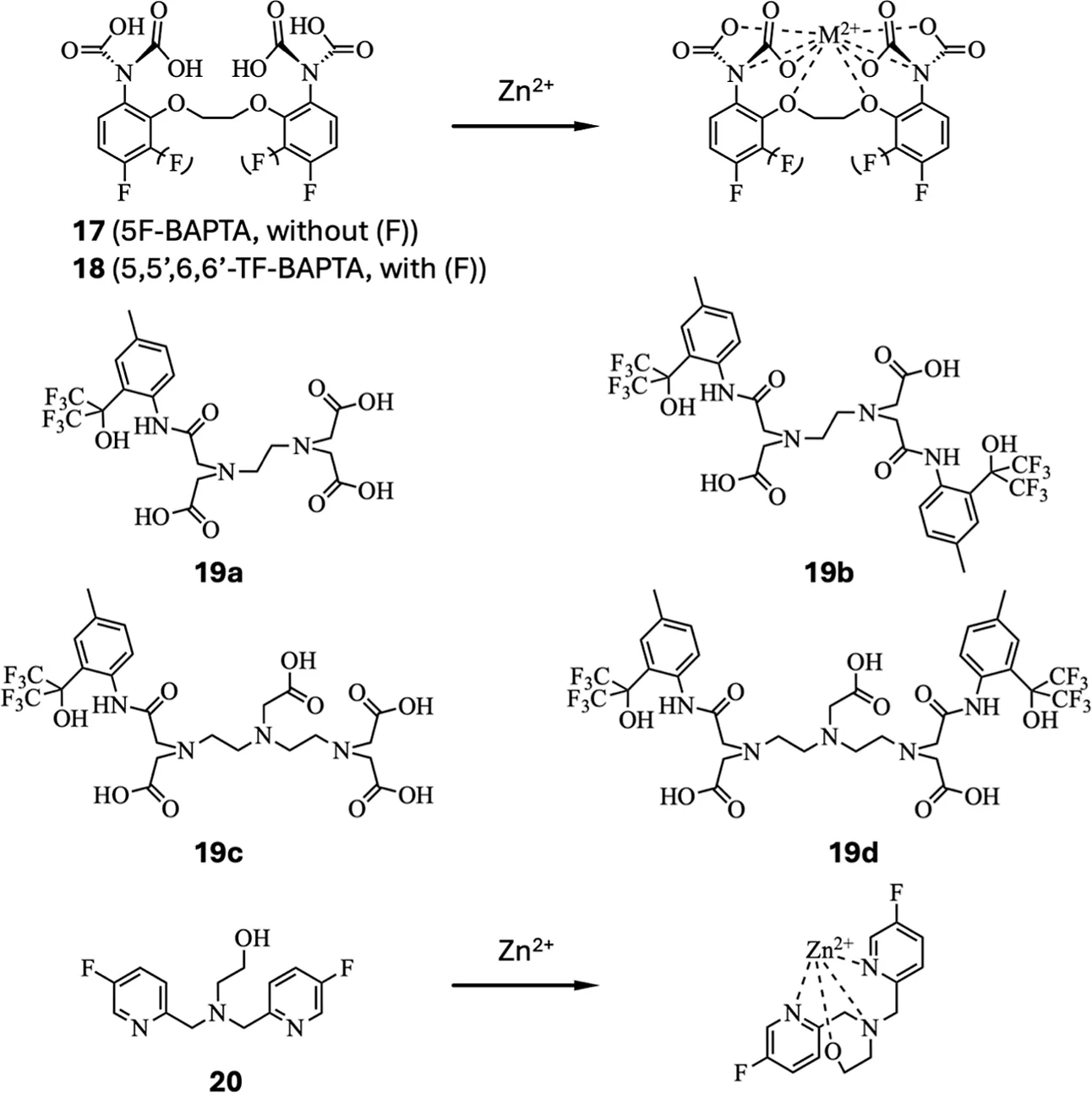
Chemical structures of fluorinated compounds utilized as zinc-specific iCEST MRI contrast agents.

**Figure 14:**
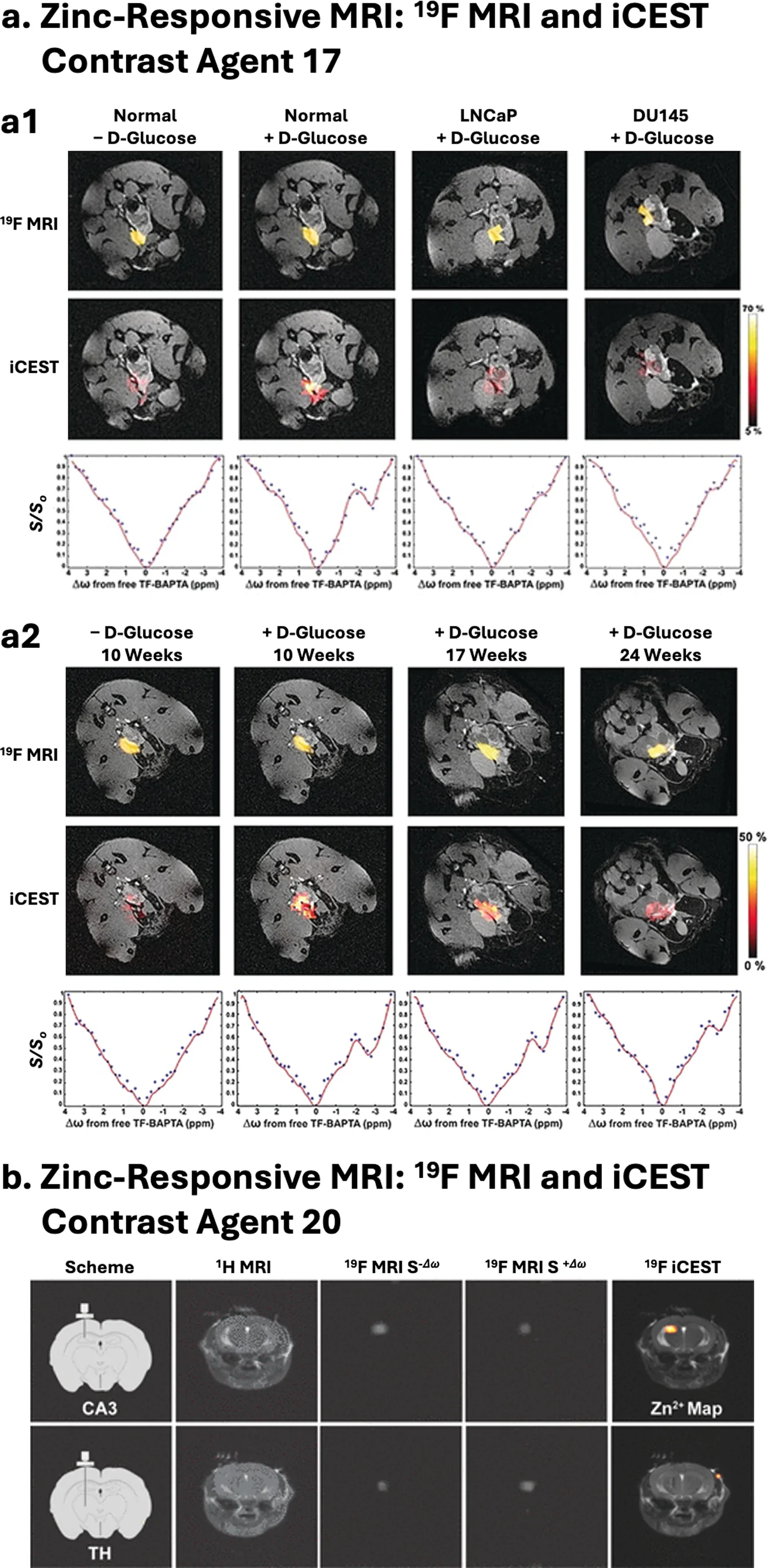
iCEST MRI imaging studies demonstrate the high sensitivity of two fluorinated zinc-specific contrast agents, **17** (TF-BAPTA) and **20**, to differentiate varying zinc levels in mouse and rat models. (a) **17** associated ^19^F and iCEST MRI images, and iCEST spectra demonstrate its ability to detect varying zinc levels in several mouse models: (a(1)) Data from NSG mice includes healthy controls (imaged before and after glucose injection) and mice bearing LNCaP and DU145 tumors (imaged after glucose injection), and (a(2)) Data from TRAMP mice includes the 10 week-old group (imaged before and after glucose injection), and the 17 week and 24 week-old groups (imaged after glucose injection). All mice were injected with 0.15 g/kg of **17** (TF-BAPTA), and GSZS was induced by i.p. injection of 80 μL of 20% (w/v) d-glucose. All measurements were performed at Δω = −2.8 ppm.^178^ (a­(1,2)) were reproduced with permission from ref 178. Copyright 2019, Wiley-VCH Verlag GmbH & Co. (b) **20** associated ^19^F and iCEST MRI images successfully map labile zinc ion pools in the rat brain, differentiating the zinc-rich Cornu Ammonis 3 (CA3) region in the hippocampus (top) and the zinc-poor region in the thalamus (TH, bottom). The images were acquired following the infusion of **20** (10 mM) at a rate of 0.25 μL/min. The final ^9^F-iCEST contrast (Zn^2+^map) was obtained by subtracting ^19^F-MRI S^+Δω^ from ^19^F-MRI S−Δω, which were produced with a presaturation pulse applied at Δω = −3.2 ppm (off-resonance) and Δω = +3.2 ppm (on-resonance), respectively. The map was then overlaid on the ^1^H MRI to provide anatomical context.^181^ Reproduced with permission from ref 181. Copyright 2021, American Chemical Society.

#### 4.2.2 Zinc-Responsive ParaCEST and Parashift MRI Contrast Agents

As illustrated by the CEST mechanism, a large chemical shift offset (Δω) between two exchanging pools of spins is highly desirable for the enhancement of the CEST signal.^171^ Involving a paramagnetic lanthanide (Ln) ion-based macrocyclic center with the paraCEST method could be an appealing strategy to explore. For example, the Sherry laboratory reported the first design of paraCEST contrast agent, **21** (EuDOTA­(gly)_4_) (Figure 15), possessing much larger chemical shift (Δω) ranging from +500 to −720 ppm relative to the resonance frequency of water.^182^

**Figure 15:**
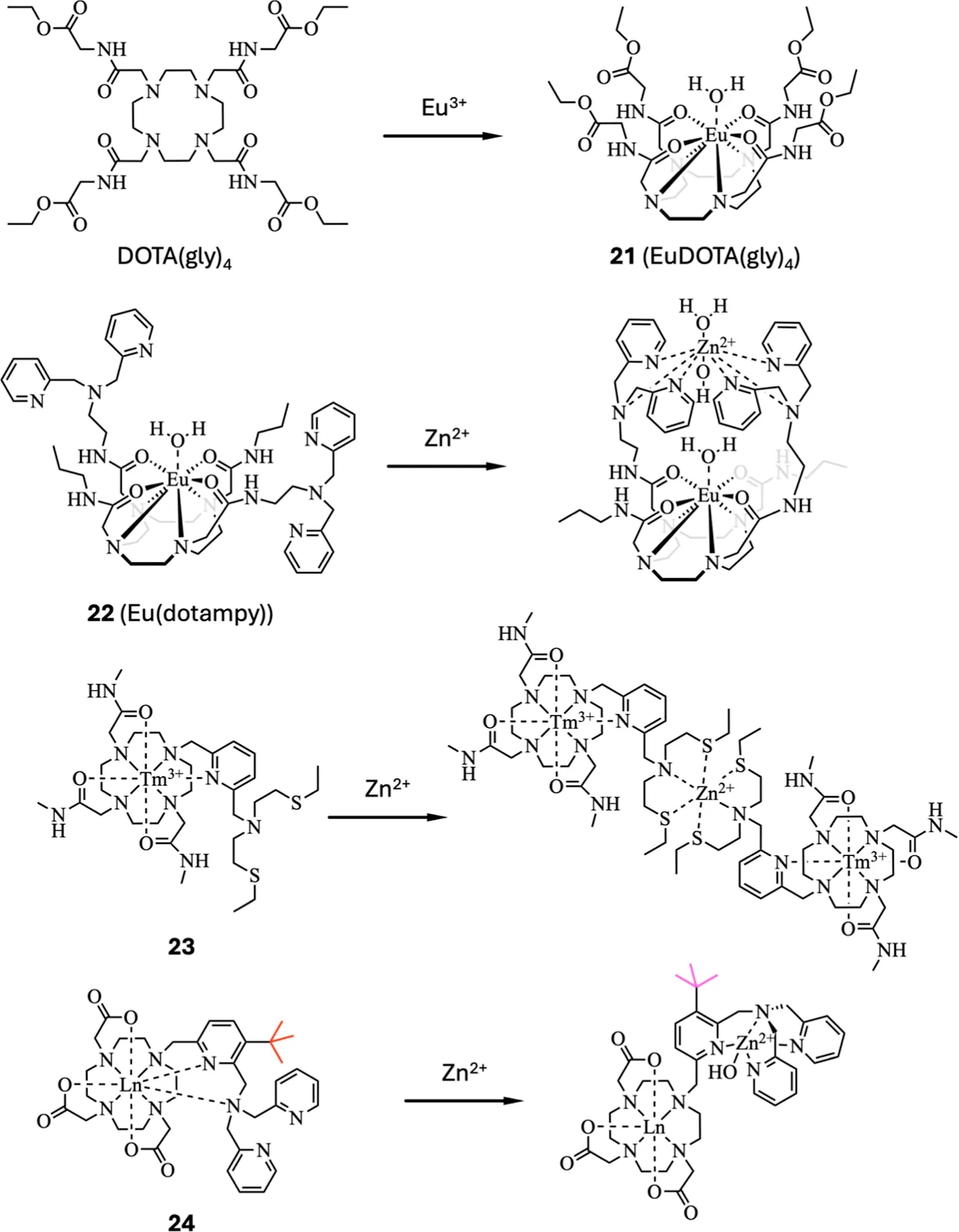
Chemical structures and contrast-forming mechanisms for paramagnetic lanthanide ion-based zinc-specific ParaCEST contrast agents (**21**, **22**, and **23**) and the zinc-specific Parashift contrast agent (**24**).

ParaCEST relies on the spin saturation exchange between the protons of inner-sphere water bound to the Ln^3+^ ion and those of free bulk water, characterized by the exchange rate (*k*_ex_), which is inversely related to the water residence lifetime (τ_m_)–the duration that the inner-sphere water remains bound to the Ln^3+^ ion (*k*_ex_ = 1/τ_m_) (Figure 8). Although paraCEST allows faster water exchange rates, *k*_ex_ must be lower than the chemical shift offset (Δω) to satisfy the critical CEST condition (Δω > *k*_ex_).^183^ The team also reported the first-generation zinc-sensing paraCEST contrast agent **22** (Eu­(dotampy)) (Figure 15). Interestingly, this contrast agent exhibits pH dependence; when coordinated with one equivalent of zinc, the signal intensity of the Eu^3+^-bound water molecules slightly decreased at pH 7.1 but disappeared at pH 8.0, demonstrating excellent sensitivity.^154^ Furthermore, Srivastava et al. synthesized Tm^3+^-based paraCEST contrast agent **23**, which turns the CEST signal from “on” to “off” in the presence of zinc due to the increased proton exchange rate between the amide group and the bulk water (Figure 15).^184^ Overall, despite the design of various novel zinc-sensing paraCEST contrasts, no in vivo zinc imaging studies have been conducted due to the complexity and high dose requirements, warranting further exploration.^168,185^

While paraCEST agents offer high sensitivity through chemical exchange, parashift contrast agents emerge as a compelling alternative for zinc sensing by leveraging pseudocontact shifts. Unlike paraCEST, which requires radiofrequency saturation pulses that are often restricted by in vivo Specific Absorption Rate (SAR) limits, parashift agents utilize paramagnetic lanthanide ions (such as Tm^3+^, Dy^3+^, or Ho^3+^) to induce a direct, “always-on” frequency shift of reporter protons. This shift moves the signal up to 100 ppm away from the bulk water peak, providing a background-free readout. Because the shift is hypersensitive to the geometric orientation, specifically the distance and angle, of the reporter protons relative to the metal center, the binding of Zn^2+^ triggers a conformational pivot that manifests as a distinct change in resonance frequency, allowing for precise, ratiometric detection without the need for external saturation. For instance, in a proof-of-concept study, Harnden et al. reported the pivotal development of parashift agent **24** (Figure 15), which consists of a modified DO3A macrocyclic ligand for coordinating a lanthanide ion, and a tris-pyridylamine (TPA) moiety for specific zinc ion binding.^186^ Coordination of the zinc ion to the TPA scaffold induces a significant conformational reorganization of the complex, altering the spatial orientation of the reporter protons. In the Tm^3+^ analog, this geometric shift results in a distinct shift of the *tert*-butyl reporter signal from −68 ppm in the zinc-free state to −78 ppm upon zinc binding. This 10 ppm frequency change enables clear, background-free, and ratiometric detection, significantly improving the clinical viability of zinc-responsive imaging.

### 4.3 Hyperpolarized Zinc-Responsive MRI Contrast Agents

Hyperpolarization in MRI represents a transformative approach to overcoming the inherent sensitivity limitations of nuclear spin systems by artificially producing a substantial, transient increase in polarization, thereby enhancing the signal-to-noise ratio of the probe by over 10,000-fold relative to thermal equilibrium. The core mechanism involves enhancing the weak thermal alignment of the Boltzmann distribution by transferring high levels of spin order from a source—such as electron spins in dissolution Dynamic Nuclear Polarization (d-DNP) and the nuclear spin isomers of parahydrogen (PHIP) for polarizing ^13^C and ^15^N, or alkali metal vapors in spin-exchange optical pumping (SEOP) of ^3^He and ^129^Xe.^187^ In the context of zinc-responsive imaging, the utilization of hyperpolarized sensors could potentially provide a unique solution to the long-standing challenge of quantifying trace metal flux in vivo. By designing zinc sensors—typically labeled with ^13^C or ^15^N—that exhibit a massive, discrete chemical shift upon zinc ion coordination, researchers can achieve precise ratiometric quantification that is independent of the absolute probe concentration. This “always-on” spectroscopic signature persists throughout the lifetime of the hyperpolarized state, effectively addressing the inherent signal-to-background limitations and concentration ambiguities of traditional paramagnetic agents. Thus, the hyperpolarization technique enables high-sensitivity mapping of mobile zinc in deep tissues, such as the pancreas or prostate, providing a level of metabolic detail previously attainable only through ex vivo or invasive biopsy techniques.

#### 4.3.1 Dynamic Nuclear Polarization Associated MRI Imaging of Zinc

DNP is the primary polarization technique under extensive investigation for creating agents to assess diseases associated with abnormal metabolism.^188^ As illustrated in Figure 8c, it utilizes microwave irradiation to transfer polarization from unpaired electron spins in radical molecules to nearby nuclei (such as ^13^C or ^15^N) in the contrast molecules at cryogenic temperatures and high magnetic fields.^189^ The polarized sample is then rapidly dissolved with a superheated solvent, producing a hyperpolarized (HP) solution with significantly enhanced the NMR signal intensity for magnetic resonance spectroscopy or imaging (MRS/MRI) studies.^190^ DNP and other nuclear polarization methods enable MRS/MRI to dynamically measure the metabolic processes of ^13^C substrates, assess the activity of relevant enzymes, and interrogate the tumoral microenvironment for cancer detection and risk stratification, offering unique functionalities not found in other imaging modalities.^191–194^ However, the technique is limited by the very short lifetime (∼2 to 3 min) of the hyperpolarized state, which rapidly relaxes toward thermodynamic equilibrium through spin–lattice (longitudinal T_1_) relaxation after dissolution and return to room temperature. As a result, the enhanced polarization is transient and must be exploited within a narrow time window.

Current DNP-associated MRS/MRI research focuses on polarizing ^13^C-labeled endogenous metabolite molecules and imaging their metabolic processes to assess tumor status, such as the predominantly investigated conversion of HP [1-^13^C]­pyruvate to [1-^13^C]­lactate.^192,195^ Thus, exploring innovative designs for ^13^C-labeled biosensors and data acquisition methods to investigate various biomarkers, such as metal homeostasis, pH,^196,197^ and redox status^191^ in the tumor microenvironment, is of great interest and significantly contributes to the hyperpolarization research community.^193,194^ For example, the studies on designing and evaluating hyperpolarized zinc-specific sensors have demonstrated the importance and innovation in this field.^155,198–200^

Mishra et al. initially explored the feasibility of using the DNP technique to enhance NMR signals of ^13^C-labeled well-known metal chelating agents, **25** (^13^C-EDTA) and ^13^C-EGTA, for detecting and identifying metal ions (Figure 16). By comparing the chemical shift difference between the hyperpolarized ^13^C NMR signals from free- and metal-bound chelators, they could identify multiple important divalent metals, including Ca^2+^, Mg^2+^, Zn^2+^, Cd^2+^, As^2+^, and Pb^2+^. Consequently, using metal-specific chemical shift imaging, the team simultaneously imaged divalent metals, such as Ca^2+^ and Mg^2+^ ions, in opaque biological samples.^198^

**Figure 16:**
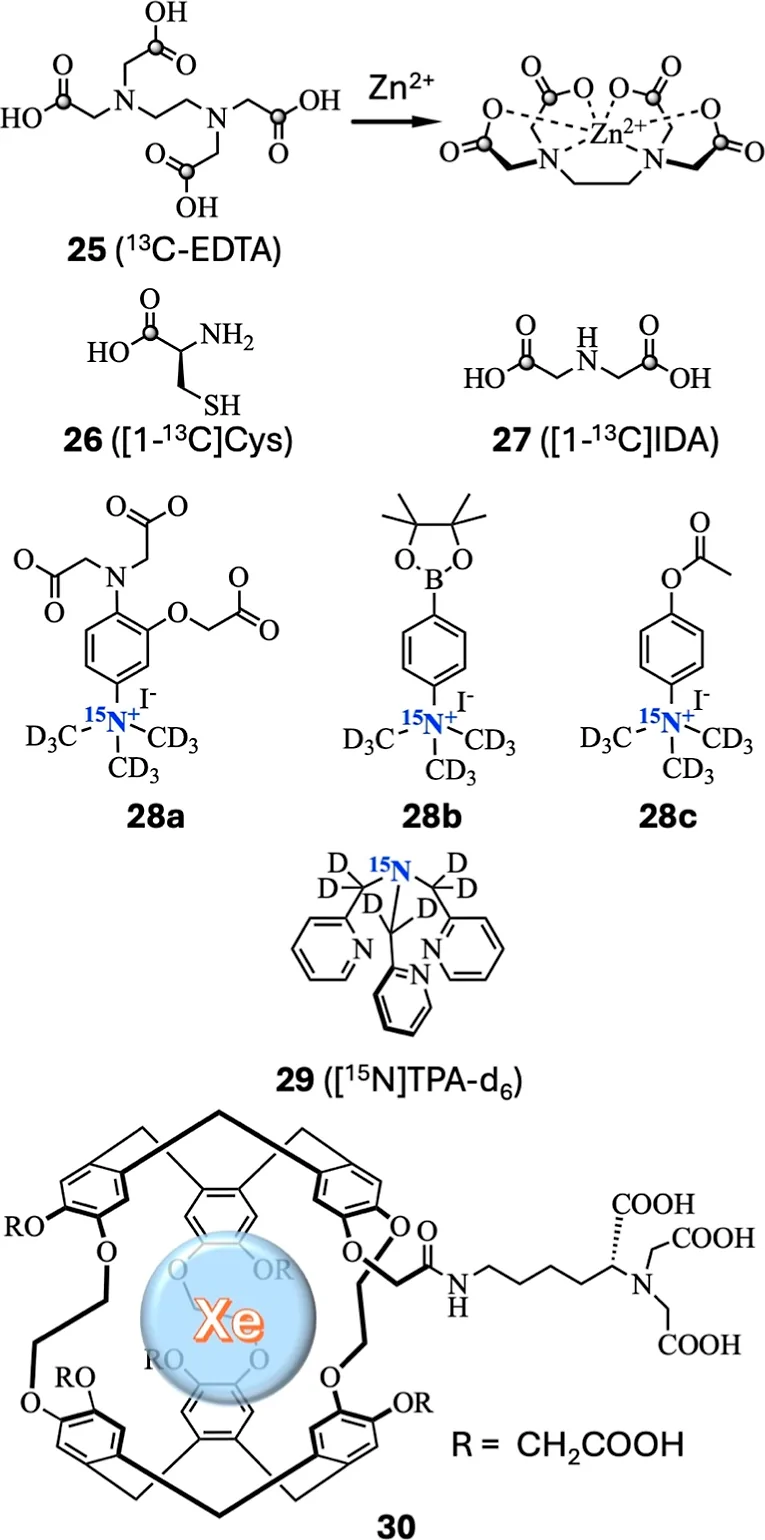
Chemical structures of zinc-specific contrast agents for nuclear hyperpolarization studies.

Furthermore, Wang et al. polarized and examined a large number of zinc-specific chelating molecules and proposed criteria for selecting optimal biosensors. Importantly, the team provided guidelines for selecting a zinc chelator based on the logarithm of the stability constant for zinc coordination (log *K*). Generally, hard-donor ligands, such as those containing hydroxyl groups, display high stability constants; when log *K* exceeds 6.9, these chelators exhibit a distinct chemical shift response upon zinc binding. Conversely, soft-donor ligands, such as pyridine-based chelators with lower stability log *K* < 5.2, do not exhibit such a response. Ultimately, the team identified **26** (l-cysteine-1-^13^C ([1-^13^C]­Cys)) and **27** (iminodiacetic acid-1-^13^C_2_ ([1-^13^C_2_]­IDA)) (Figure 16) as effective zinc biosensors with sufficient chemical shift differences upon zinc binding. These biosensors demonstrated the ability to target and specifically image zinc ions in biological samples, distinguishing them from other endogenous analytes and allowing for quantitative determination of zinc concentrations in both phantoms and biological samples.^199^

Although less frequently reported than ^13^C-enriched biosensors, DNP can also polarize ^15^N-labeled molecules, providing several advantages, including narrow line widths, long *T*_1_ relaxation times, a broad chemical shift range of 900 ppm, and minimal in vivo background interference. For example, Nonaka et al. established a ^15^N molecular platform, **28** ([^15^N, D_9_]­trimethylphenylammonium) (Figure 16, **28a–c**), with an exceptionally long *T*_1_ relaxation time (816 s at 14.1 T), on which versatile structures were developed for various applications, including sensing calcium ions, reactive oxygen species, and enzyme activity.^201^

Regarding zinc-detecting HP ^15^N MRI/S sensors, Suh et al. synthesized and polarized **29**, a ^15^N-labeled and deuterated tris­(2-pyridylmethy)­amine ([^15^N]­TPA-*d*36) scaffold (Figure 16),^55^ which is a zinc-targeting structure with excellent membrane permeability.^118^ This sensor displayed a significant ^15^N NMR chemical shift (20 ppm) between the free sensor and the zinc–bound complex, with substantially long T_1_ relaxation times of 70.9 ± 1.1 s and 57.0 ± 2.3 s at 9.4 T, respectively. Furthermore, in vitro imaging demonstrated this sensor can effectively quantify free zinc in homogenized human prostate tissues and intact cells.

#### 4.3.2 ^129^Xe Spin-Exchange Optical Pumping Associated Zinc Sensing MRI Imaging

In addition to DNP, SEOP emerges as a cutting-edge technique for polarizing the noble gas xenon-129 (^129^Xe), facilitating ^129^Xe MRI for human lung imaging.^202^ Kotera et al. innovatively designed a cryptophane core to eencapsulate ^129^Xe, which was tethered to a zinc-specific binding moiety nitrilotriacetic acid (NTA) to construct sensor **30** (Figure 16).^200^ Zinc binding to NTA constrains the flexibility of the linker, resulting in a downfield shift in the ^129^Xe NMR signal that enables zinc detection and MRI imaging. Although this study successfully demonstrated the potential of SEOP for zinc imaging, its practical implementation in vivo may encounter considerable challenges.

## 5 Summary

Zinc ions play a critical role in various physiological processes in vivo, with their distribution and trafficking tightly regulated to maintain normal biological functions. Disrupted zinc metabolism, reflected as changes in zinc concentrations and spatial distribution, has been recognized as a valuable biomarker for the detection and stratification of various aggressive and prevalent diseases, such as cancers, central neurological disorders, diabetes, and osteoarthritis. High-resolution optical imaging, particularly fluorescence microscopy, has been pivotal for studying cellular and subcellular zinc dynamics, providing detailed insights into zinc compartmentalization, transport, and signaling. These methods enable the mapping of zinc pools within organelles, monitoring of rapid zinc fluxes in response to stimuli, and elucidation of zinc-dependent regulation of enzymes, transcription factors, and ion channels. Furthermore, optical techniques have revealed disease-associated disruptions in zinc homeostasis and facilitated the development of novel zinc-sensitive probes, offering a direct link between molecular-scale zinc dynamics and cellular functions.^21,24^ However, understanding how cell-specific zinc metabolism integrates with systemic organ function, especially under pathological conditions, remains an important area of investigation. The development of clinical imaging technologies capable of noninvasively imaging alterations in zinc homeostasis and correlating them with disease status helps bridge this critical knowledge gap between cellular-level pathology and organ-level changes, and is essential for advancing precision medicine.^203^ Currently, research efforts are primarily focused on the proof-of-concept stage, with limited in vivo evaluations. Given the complexities of both chemical sensor structures and biological systems, validating the efficiency of these sensors through in vivo studies is imperative before concluding on their success. To achieve this, extensive research has focused on developing diverse zinc biosensors and associated imaging modalities, broadly categorized into radionuclide imaging (e.g., PET and SPECT) and nuclear magnetic resonance imaging (MRI).

MRI offers distinct advantages in spatial and temporal resolution over radionuclide imaging. Nevertheless, its intrinsic sensitivity is comparatively lower than that of nuclear or optical imaging techniques, indicating the necessity for sufficiently high *r*_1_ values in the development of MRI-based sensors. The extrapolation of *r*_1_ values from in vitro experiments to in vivo performance is often tenuous. A case in point is the relationship between the *r*_1_ value and the MRI contrast responsive to zinc, which is not consistently correlated, as it depends largely on the actual in vivo biological context.^145^

Given the inherent limitations of MRI in terms of sensitivity for molecular sensing, radionuclide imaging, particularly PET, offers significant advantages for detecting zinc in vivo. Its paramount strength lies in its high sensitivity for detecting picomolar to nanomolar concentrations, which is crucial for tracing zinc at low physiological levels. This high sensitivity is also complemented by inherent quantitative capabilities, allowing for precise measurement of zinc concentrations in tissues and providing invaluable insights into its kinetics and distribution. Furthermore, the deep tissue penetration of PET’s 511 keV γ rays enables whole-body, noninvasive monitoring of zinc, offering a distinct advantage over optical techniques. This noninvasive nature facilitates repeatable dynamic studies of zinc trafficking in living organisms. Finally, PET’s ability to provide molecular and functional information through specific radiotracers, coupled with its established translational potential in clinical settings, makes it a powerful and indispensable tool for understanding zinc’s complex roles in health and disease.

In conclusion, zinc imaging research is at an exciting juncture, poised to deliver transformative insights. The ongoing innovation in probe design, coupled with advancements in imaging technologies and a deeper understanding of zinc biology, holds immense promise. This synergy is critical for illuminating the fundamental roles of this vital micronutrient in health and disease, ultimately paving the way for the development of novel diagnostic tools and more effective therapeutic strategies across a wide range of conditions. As these technologies progress, we can anticipate a more insightful and dynamic view of zinc’s involvement in everything from neurological function to immune responses, fundamentally changing how we understand and address numerous pathologies.

## Vocabulary

Zinc Homeostasis: The cellular zinc levels are tightly regulated by ZIP transporters (influx), ZnT transporters (efflux), and zinc storage proteins to maintain essential physiological functions in vivo. When this system is disrupted, it can lead to systemic pathologies, including neurodegeneration, chronic inflammation, and metabolic disorders.
Zinc-Associated Diseases: Disorders in which disrupted zinc homeostasis contributes to disease development or progression, including neurodegeneration, diabetes, inflammation, and certain cancers.
Nuclear Imaging: A medical imaging technique that uses radioactive tracers to visualize and quantify physiological and molecular processes in vivo, providing insight into organ function, metabolism, and disease states. Common modalities include Positron Emission Tomography (PET), which detects positron-emitting isotopes, and Single Photon Emission Computed Tomography (SPECT), which captures gamma-ray emissions to produce three-dimensional images.
Radioactive Zinc Ions: Radioactive isotopes of zinc—primarily ^65^Zn, ^63^Zn, and ^62^Zn—used as powerful radiotracers to map zinc absorption and distribution within biological systems. ^65^Zn emits γ radiation, while ^62^Zn and ^63^Zn are positron emitters suitable for PET imaging, enabling quantification of metabolic flux and identification of zinc homeostasis disruption in tissues.
Zinc-Specific Nuclear Probes: Molecular probes labeled with radioactive isotopes that detect and track zinc in vivo, allowing visualization of zinc distribution and dynamics using nuclear imaging techniques such as PET or SPECT.
Magnetic Resonance Imaging (MRI): MRI is a noninvasive technique that uses high-intensity magnetic fields and radiofrequency pulses to resolve internal anatomical structures. Beyond morphology, it facilitates molecular-level characterization, enabling the detection of specific ions, metabolites, or contrast agent kinetics for integrated anatomical and functional analysis.
Zinc-Responsive MRI Contrast Agents: MRI contrasts that change their signal in response to zinc binding enable in vivo visualization of zinc distribution and dynamics.
